# On reappearance and complexity in musical calling

**DOI:** 10.1371/journal.pone.0218006

**Published:** 2021-12-17

**Authors:** David M. Schruth, Christopher N. Templeton, Darryl J. Holman

**Affiliations:** 1 Department of Anthropology, University of Washington, Seattle, Washington, United States of America; 2 Department of Biology, Pacific University, Forest Grove, Oregon, United States of America; University of Missouri Columbia, UNITED STATES

## Abstract

Music is especially valued in human societies, but music-like behavior in the form of song also occurs in a variety of other animal groups including primates. The calling of our primate ancestors may well have evolved into the music of modern humans via multiple selective scenarios. But efforts to uncover these influences have been hindered by the challenge of precisely defining musical behavior in a way that could be more generally applied across species. We propose an acoustic focused reconsideration of “musicality” that could help enable independent inquiry into potential ecological pressures on the evolutionary emergence of such behavior. Using published spectrographic images (*n* = 832 vocalizations) from the primate vocalization literature, we developed a quantitative formulation that could be used to help recognize signatures of human-like musicality in the acoustic displays of other species. We visually scored each spectrogram along six structural features from human music—*tone*, *interval*, *transposition*, *repetition*, *rhythm*, and *syllabic variation—*and reduced this multivariate assessment into a concise measure of musical patterning, as informed by principal components analysis. The resulting *acoustic reappearance diversity index* (ARDI) estimates the number of different reappearing syllables within a call type. ARDI is in concordance with traditional measures of bird song complexity yet more readily identifies shorter, more subtly melodic primate vocalizations. We demonstrate the potential utility of this index by using it to corroborate several origins scenarios. When comparing ARDI scores with ecological features, our data suggest that vocalizations with diversely reappearing elements have a pronounced association with both social and environmental factors. Musical calls were moderately associated with wooded habitats and arboreal foraging, providing partial support for the acoustic adaptation hypothesis. But musical calling was most strongly associated with social monogamy, suggestive of selection for constituents of small family-sized groups by neighboring conspecifics. In sum, ARDI helps construe musical behavior along a continuum, accommodates non-human musicality, and enables gradualistic co-evolutionary paths between primate taxa—ranging from the more inhibited locational calls of archaic primates to the more exhibitional displays of modern apes.

## Introduction

Acoustic display behavior, including song-like and musical calling, has independently evolved in several vertebrate [1] and some arthropod [2] clades. However, the historical selection pressures that gave rise to this behavior, and its current adaptive function, are not well understood. Ascertaining the factors that might have driven the emergence of human music, for example, is difficult due to its auditory transitoriness and a scarcity of paleontological artifacts—although some fossil musical instruments have been discovered [3]. Consequently, we have few clues available to resolve if human music is a unique human adaptation or simply an evolutionary continuation of the musical calls of gibbon-like primates [4, 5], and if it has any current adaptive value [6]. As an alternative to more typical human-focused or archaeological approaches, biomusicologists can investigate ultimate evolutionary functions and mechanisms across animal taxa by using behavioral data from extant organisms [7, 8]. Adopting this methodological approach, we entertain Darwin’s original notion of a pre-human proto-musicality [5] in considering the possibility that acoustic utterances evolved gradually from unexceptional animal communication signals into something more akin to human music [9].

A number of functions have been proposed for the evolution of complex acoustical behavior ranging from social display signaling theories [5, 10–13] to habitat positioning oriented ones [14–17]. One of the latter of these animal communication based theories, the acoustic adaptation hypothesis (AAH) [14, 18, 19], posits that animals should evolve particular spectral features for improved transmission of a signal through the unique acoustic setting (e.g. vegetative obstruction) of a given habitat. For example, AAH predicts that low frequency vocalizations should correlate with high vegetation density [18]. This has been previously demonstrated in primates [19]. A version of AAH further predicts that there will be more inter-element intervals as vegetation structure becomes more complex [18]. Habitat based theories such as AAH, have been modestly supported by animal communication research over the years [20], but recent studies on bird song have found only weak support [21, 22].

Theories focusing on social functions of musical behavior, deriving almost entirely from the human music origins literature, can be arranged on a continuum spanning both intra- and inter-group directed calls [23, 24]. These include emotion regulation [25], language acquisition [26, 27], emotional communication [28, 29], social (e.g. pair) bonding [4, 24], sexual advertisement [5, 13], acoustic defense [30], group cohesion [25], group selection [31], and coalition signaling [11]. While social and habitat factors likely both play a role, we suggest that each of these approaches typically neglect illumination of a zoologically broad solution to the mystery of function because they lack a generic, mathematical, and acoustic-features based definition of musical utterance.

The existing definitions used in these various origins theories understandably struggle with a seemingly unavoidable circularity [7] and lack of consensus regarding theoretical foundations [32, 33]. Indeed, objectively defining *song*, *music*, or even the more ecumenical term *musicality*, has proven to be surprisingly challenging [6]. Some notable attempts include: *music* as “the art of combining tones” [34], “an abstract pattern of sound” [35], an emotionally motivating and information rich “holistic pattern” [25], “a type of social reward system” [31], “embodied expressive movement” [36], or as verbs “formalization, repetition, elaboration, exaggeration” [35] and “embodying, entraining, and transposably intentionalizing time in sound and action” [37]; *song* as “elaborate, [loud and complex] patterns of vocalization” [4], “truly creative[,] orderly, organized, structured [sequences with] repeatable distinctive patterns” [38], “complex learned vocalization” [7], sounds that are “for the most part pure in tone and musical in nature” [39], “rhythmically and/or melodically organized [speech]” [40], or “a complex combination of notes” [41]; and *musicality* as a spontaneously developing [music] production and processing capacity constrained by neurobiology [42, 43].

Our main objections with the above definitions centers upon two main issues, first that many circularly retreat to similarly elusive words such as “complexity” or “musical,” and secondly that they tend to remain stubbornly confounded in the trappings of an originating context. While broad binary definitions are canonical for theories on the social origins of music [11], theories on habitat selection for elements of song tend to, instead, be overly constrained to specific features, such as fundamental frequency [19, 21, 22]. But music is notably appreciated for its component modularity [6, 40, 42, 44, 45] and the combinatorical composabilty such modularity enables. Very few studies to date have successfully attempted to multiplicatively combine these acoustically narrow structural features [41, 46] to objectively encompass a zoologically broad essence of musical display.

Although many have sought to understand how specific evolutionary forces act on specific acoustic features of musical signals [6, 13, 42, 47], many only study *listeners* [11, 13, 45], usually of western music, a culture where consumers vastly outnumber producers [48], and only a minority have focused on features of musical *performance* [32, 49]. Correspondingly, we have purposefully underutilized universals studies [46, 50–52] focusing on the audio perceived [53] by the *receivers* (e.g. pitch [16]), in order to capitalize instead upon the vocal signals, or “the sound itself” [54], produced by the *senders* (e.g. tone). We endeavored to construct a taxonomically all-encompassing, and potentially more ancestrally enlightening, formulation of these structural acoustic features, the signal itself, informed by those present in avian song and rooted primarily in those that are exceedingly universal to human music [55].

To further aid in developing this more liberal characterization of musical calling, we also distinguish utterance level features—those present in every *piece or performance*—from conserved or common features—those present to some degree, “most if not all” [9], in a musical *system or culture* [34]. Non-vocal modes of generation (e.g. via instruments) and cultural musical contexts (e.g. dance and rituals) are common human *system-level* universals [56], but they are rare in other vertebrates. Furthermore, a context is not inherently acoustic itself [41] and might best be reconsidered as a co-evolutionary *influence* on acoustic display. Musical contexts, including learning and other evolutionary cofactors, can instead be tested later as potential influences on this independently constructed acoustic-based index. Accordingly, we focus our initial index development efforts only on *structural* acoustic universals (e.g. pitch, melody, and rhythm) of human music [9, 56] possibly corresponding to biologically evolved components of underlying musicality (e.g. production and processing for tone, interval, and meter) [57].

A balanced investigation that considers both a broad universality [58] in combination with specific acoustic features seems most promising [55]. That is, acoustic features that are common to both human music as well as more elaborate animal calls could be considered biologically ancestral and deriving from similar evolutionary pressures [57, 58]. While we hypothesize an evolution by ecological homology, we abide by the null that musical calling could have randomly emerged in various independent clades as a coincidental convergence. Here we attempt to bridge this gap between the notion of an exclusive, human-only claim on musical behavior and the reality that the many animals’ complex calls (e.g. those of orthoptera, sphenisciformes, pinipeds, aves, cetaceans, and primates) could qualify as “musical” utterances, even by our anthropocentric human standards [5, 16, 38, 59–61]. Extremely complex utterances—such as linguistic speech and music (e.g. time signatures and musical keys)—tend to be uniquely human, but here we instead explicitly focus on the simpler, but essential, underlying components of such higher-order musical complexity.

Contrary to the notion of a strict, categorical boundary between the musical and non-musical, we make the case here (akin to what others have done for “musilanguage” [62] or the vocal learning continuum hypothesis [63]) that musical behavior is a spectrum phenomenon that likely gradually emerged from (or into) other, related vocal behaviors [64]. Our approach differs from previous work [1, 65], in that definitional features need not be uniquely human [57], but should be generic enough to be exceedingly prevalent amongst, if not obviously ancestral to, all humans. While we acknowledge that all universals are at some level statistical, probabilistic, and non-absolute [9, 66], we suggest here that those present at smaller time-scales (e.g. unit repetition and intervalic frequency change) tend to be more broadly applicable across cultures and species. We subscribe to the view that the most productive way to encompass Darwin’s zoologically broader notion of acoustic musicality [5] is to explore these more modestly essential components of human music and ask whether they might also apply to calls of various non-human animals [67].

**Fig 1:**
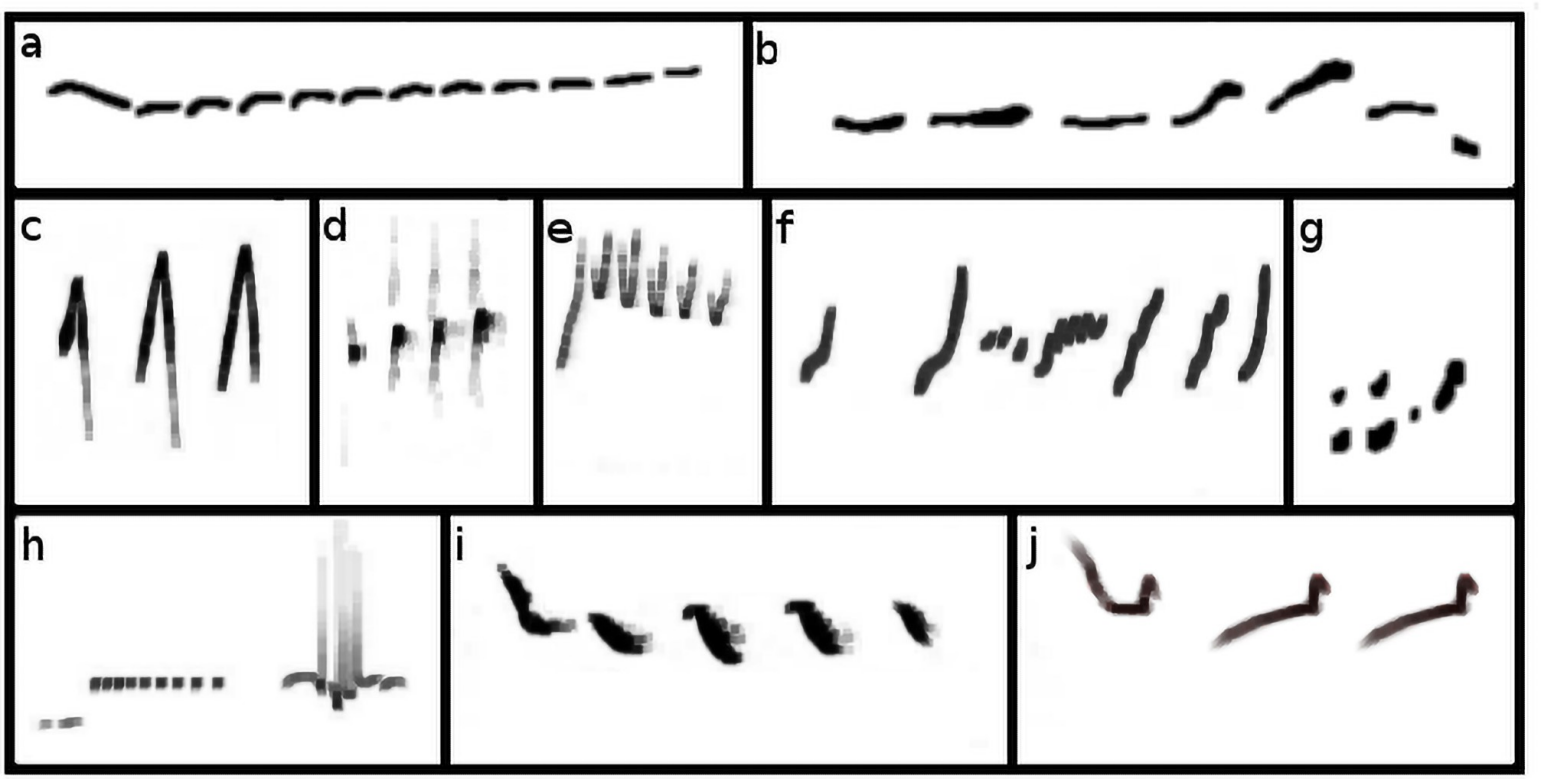
Various spectrographic reproductions of music-like primate calls. Spectrographic representations of ten species’ calls with corresponding max *acoustic reappearance diversity index* (ARDI) scores formulated as syllables × (P(repetition) + P(transposition)). **a**: female scream #773 of *Tarsius spectrum* 2.6×(0.54+0.56) = 2.8, **b:** female great call #246 of *Hylobates agilis* 4.6×(0.5+0.2) = 3.3. **c**: location trill #649 of *Saimiri sciureus* 1.4×(0.7+0.16) = 1.23, **d**: tsic #190 of *Galagoides demidoff* 1.6×(0.54+0.58) = 1.8, **e**: twitter #726 of *Tarsius syrichta* 2.2×(0.38+0.54) = 2.0, **f**: quaver interlude #254 of *Hylobates lar* 4×(0.6+0.3) = 3.5, **g**: musical sequence #363 of *Macaca fuscata* 2.2×(0.4+0.2) = 1.3, **h**: male short phrases #861 of *Hylobates leucogenys* 4.6×(0.74+0.1) = 3.9, **i**: modified twitter hook (593) of *Saguinus fuscicollis* 2.4×(0.6+0.16) = 1.8, **j**: alerting call of #88 *Cebuella pygmaea* 1.8×(0.6.+0.1) = 1.26. Many of these short calls have high degrees of transposition given their brevity. Such marked inter-element intervals likely suit callers for acoustic adaption to arboreal environments. See S1 File reference list for spectrograph sources.

Structural acoustic features that are exceedingly prevalent at small durations in human music also share substantial overlap with many features of bird song [51]. Unlike research on human music origins, which predominantly tends to focus on rhythmic universals, birds’ mate choice preferences for performance and complexity [68] has canalized avian bioacoustics research towards a focus on more spectral aspects of display such as frequency variation and syllabic diversity [69]. Analysis of bird song can entail visual quantification of aesthetic features of possible signaling importance present in spectrograms—plots of spectral energy over time (Fig 1). Various features present at smaller time durations that can be readily observed in spectrograms of these songs—unit *consistency* [70, 71], *trill rate* [72], *repertoire size* [69, 72–74], song *bout length* [75], and *complexity* [10, 74, 76, 77]—converge nicely with our set of utterance level human universals. While defining the somewhat opaque terms associated with the underlying components of acoustical display aesthetics (in particular, *complexity*) can itself be intimidating, recent advances in fields outside of comparative musicology can assist us in proceeding with an attempt [9]. Complexity, a short-hand term used in bird song analysis synonymously with syllabic *diversity* [74], is a useful catch-all measure, but it rarely emphasizes structural regularities such as syllabic *similarity*, and only then as repetition rather than transposition.

**Fig 2:**
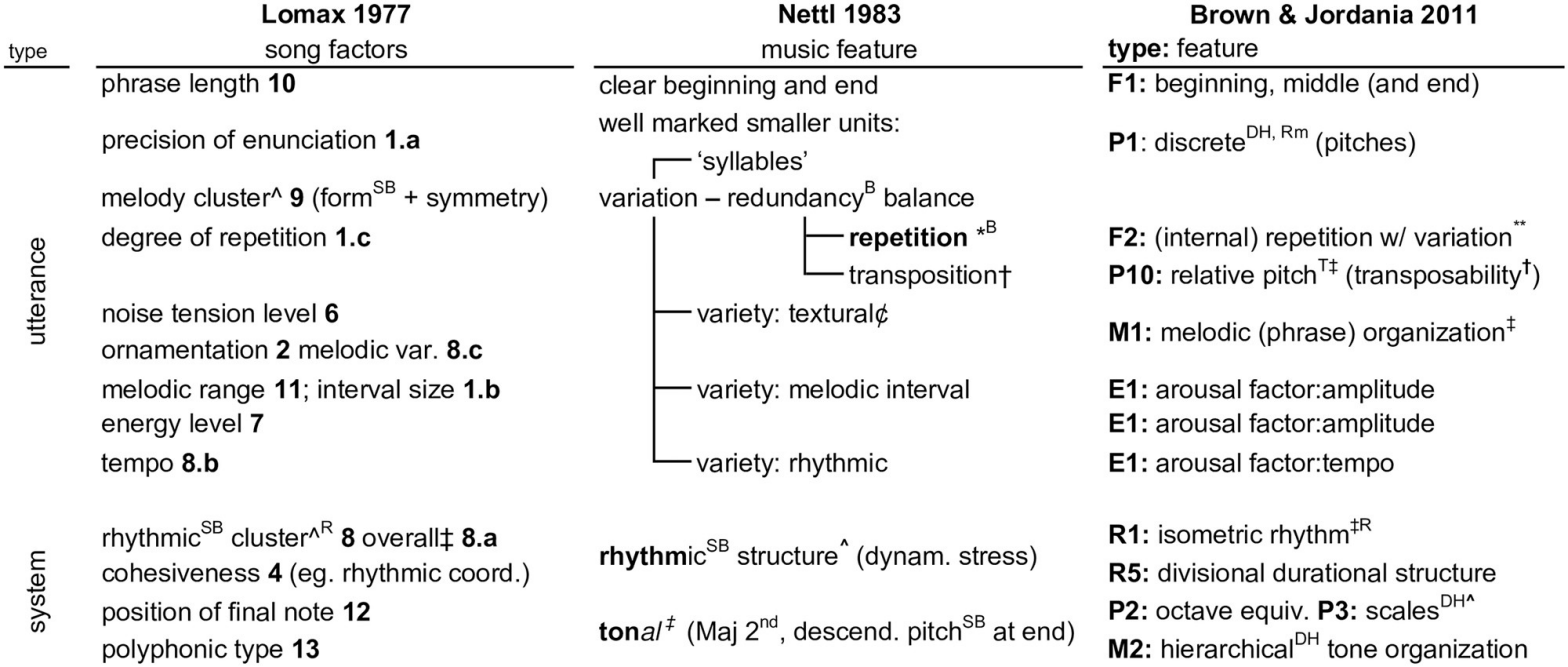
Seminal works on human musical universals and candidate structural features considered in formulating our index. Putative structural acoustic features are organized into utterance and system levels [34]. Bolding in Nettl’s column indicates the terminology we adopted for use in this study. Abbreviations in Brown & Jordania’s column indicate: P **=** pitch**,** F **=** form**,** M **=** melody**,** E **=** emotional/arousal factor, and R = rhythm (numbers indicate rank) [56]. Savage’s column lists empirical frequency-based ranks *(bolded numbers*) of universals empirically derived [45] from the previously developed “CantoCore” structural characters (italics) of song [41]. Song universals from Lomax’s study [79] (far left) and Savage’s statistical/system level focused universals (far right) were merged into the table, post-hoc, for comparison purposes; Other universals studies are superscripted into the table above as follows–B: Donald Brown (1991) human universals; DH: Dowling & Harwood (1986) psychological “tonal scale systems” [80]; ^: Carterette & Kendall (1999) [81]; T: Trehub 2000 (plus sIFR infant music) [82]; R: Ravignani (2016) rhythmic universals [83]; Rm: Ravignani melodic universals [84];*: Trehub 2015 notes the ubiquity of repetition [85]; **: Richman 2000 [86] and Mache 2000 [55] also highlight repetition while †: Cross 2001 highlights transposition; SB: Savage and Brown 2013 [9]; ‡: signaler side adaptations of Honing 2018’s [57] perception-based list of candidate core components of musicality.

Terminology differs between the academic fields that study human music and avian song, but many of the aesthetic features from both seem to group neatly into two broader categories—both a similarity between *and* diversity among units. These can be assessed, for example, by measuring the consistency *and* number of repeated units, respectively. These more simplistic melodic and form related elements (esp. repetition) might more appropriately be grouped at the broad *utterance* level of acoustic musicality. Human musical utterances have been described as consisting of multiple, discrete units (e.g. notes, chords, phrases) [9] that both vary (in pitch, tempo, or texture) and repeat [34, 56] (Fig 2: utterance). There is disagreement, however, as to whether pitch, a constituent of tone [78], and rhythm, are required features at this more basal scale of musical organization (Fig 2: system). The less common system-level universals of rhythm and tone (e.g. pitch), may not be as efficient at explaining more diverse aspects of proto-musicality. Whereas rhythm and pitch are important parts of human music and appear frequently in animal song, they may not be universal features relevant to all patterned vocal utterances of animals that exhibit music-like behavior.

To help better understand this zoological enigma, we developed, using exploratory methods, an impartial formulation of manifest musicality by collecting spectrograms of vocalizations from 55 primate species and scoring them along six musically relevant acoustic parameters—at both the *utterance* and *system* levels. We performed a principal components analysis (PCA) informed variable reduction on these six acoustic feature scores. The contrasting utterance-level features of syllabic diversity and reappearance were retained and combined into a univariate measure of proto-musicality that detects musical patterns from any acoustic utterance. The resultant *acoustic reappearance diversity index* is defined as the average number of melismatic “syllables” (i.e. unique spectral shapes) that reappear, either by repetition or transposition, within a call. We believe that this novel acoustic musicality index could be applicable to human music as well as the many other forms of animal song and music-like behavior. We demonstrate the utility of this metric by applying it to key ideas from the two bodies of origins theories mentioned above—both adaptation to habitat acoustics and selection based on social influences, showing that both are an important part of a larger and gradualistic evolutionary progression.

## Materials and methods

### Vocalization data collection

As an alternative to analyzing raw audio recordings, which are often inaccessible [9], we used published spectrograms: plots of acoustic energy where *x* = time and *y* = frequency (Fig 1). We sampled spectrographic studies from nearly all families in the primate family tree, where each vocalization collection was individually culled and classified by primatologists focusing on select species. We primarily focused on collecting *continuous* data from spectrographic vocalization repertoires (for 62 species), and only secondarily on *categorical* call type data (e.g. *loud call*, *long call*, *chorus*, *song*, *duet*) from text descriptions of vocalizations (for 199 species) [19, 87]. The spectrographic studies focused on individual species and were all published in English before 2014. The categorical data (e.g. name, type, and context) were additionally used to verify the multivariate analysis on the variables derived from the spectrographic dataset.

For the continuous, spectrographic analysis, we searched for publications meeting the above criteria by querying online search engines—initially via ISI Web of Science [88] and subsequently via Google Scholar [89]—to locate these vocal repertoires for the quantitative scoring analysis. We used “vocal* AND repertoire* AND [primate genus]” as an all-field query in Web of Science. Searching within each genus was discontinued after a sufficient number of species from each were obtained.

In general, studies were catalogs of individual species behavior rather than developmental, experimental, or species comparative studies. For each species studied, articles had to include spectrographic depictions for multiple calls, in order to obtain a variance estimate of each species’ song index. A primary objective was to obtain “complete repertoire” studies and, as a result, over 2/3^rds^ of accepted studies had more than 10 different calls (*n* = 45 species). Some exceptions were made for species with (an) obvious, stand-out display call(s) (e.g. gibbon songs) that were otherwise relatively non-vocal (*n* = 5). Some other exceptional non-repertoire focused studies (e.g. long calls, loud calls) were also included (*n* = 5). Because the main goal was to let structural acoustic features predict musical calls independent of researcher call designation, we did not include any other studies on just a single call type (e.g. contact, food, alarm). A single study (Harcourt 1993) that was neither a full-repertoire nor a loud-call study on the “close calls” of the gorilla was used because no study with a larger variety of calls was found.

We scanned 61 books and downloaded 67 PDFs to obtain spectrographic vocalizations from more than 80 species and from over 300 total leads on possibly relevant studies. Only a single spectrographic study for each species was used in the dataset, so that some studies, which redundantly covered an already collected species, were removed. In these cases, we retained the publication that described more vocalizations, included more modern recording and analysis tools, higher quality spectrograms, more sophisticated call classification techniques, or ones that were more recently published. The final collection of spectrograms was extracted from 58 sources resulting in 1,297 different spectrograms for 61 species representing 40 genera.

For 44 studies in electronic format, images were obtained as screen captures at 100% zoom. For the remaining species, we scanned spectrograms from printed articles at 300dpi as grayscale 8-bit depth bitmaps to provide similar resolution. We also used image editing software to manually clean and standardize the spectrograms by removing axes, labels, and any annotative markings. Careful effort was made to avoid truncating any features of calls that were not already constrained by the plot margins as delineated by the original authors. Vocalizations were grouped into 842 species-specific note, phrase, and song types as assigned by the original authors themselves. Ten vocalizations (from three different studies) did not meet the minimal study acceptance criteria above, leaving 832 scored vocalizations (corresponding to 1287 spectrograms from 55 sources).

We included as separate vocal types both single unit and repeated unit vocalizations, if the primary authors had also done so. Thus, most sampling biases or unit of comparison incongruities arguably derive from data collection truncation decisions made by the primary researchers. Admittedly, however, since chimps and bonobos, for example, exhibit extraordinary levels of communicative combinatorics [90, 91], the call complexity scores we determine may be underestimates—as all manifest combinations of vocal units might not be publishable under many existing journal formats.

### Spectrogram scoring

We used simple human music universals [34, 56] and the principles of acoustics [92] to guide us in selecting a total of six structural features as scoring parameters (Fig 2). These, along with spectrographic interpretations of definitions used are *tone*: the presence of clean harmonics with distinct, horizontally-parallel bands; *interval*: a sloping, jagged, or curving, rather than static, fundamental frequency (intra-element); *rhythm*: a regular recurrence or pattern of units over time; *repetition*: similarity in units repeated across time; *transposition*: (inter-element) similarity in units of different frequencies (and at different times); *variation*: number of distinct unit types or shapes within a call. Observers were trained for one hour on feature definitions [93, 94] and how to identify and quantify them spectrographically using bird song spectrograms (S1 Fig). These song feature definitions were subsequently verified using additional encyclopedia and dictionary entries [95, 96]. Manual scoring was performed over the course of two afternoons, blindly without reference to the species. For each of the six features of human music, vocalizations were scored (less than one minute per call) in a globally randomized order. Each of the six features was scored on a scale of 1 (lowest) to 10 (highest), except for variation which was scored as a count of unique syllable shapes (S1 Fig). The scoring protocol is detailed in the supplement as well as online [97].

These matrices of ordinal scores were then averaged [98] across the individual scorers to create a single 832 (vocalization) by six (feature) matrix. Scores were then rescaled to continuous (0–1) probabilities by dividing by 10. Finally, for the PCA analysis, these scores were standardized to unit variance so that each factor had an equal chance of contributing to the overall variance [99]. For comparison purposes we used not only the first principal component [PC1] and the raw syllable count [77], but a euclidean distance based song complexity index [100] as $SCI=n\times \sqrt{l^{2}-\sum {\left(m_{i}-1\right)}^{2}}$ where *n* = syllables, *l* = units, and *m*_*i*_ = repetition for each syllable. Because our repetition data did not reach the syllable-level, we used an approximation of *m* as follows. Using our call-level measures we reformulated, via an estimation for *m* as the ratio of (repeated [r]) units to (repeated) syllables, E(*m*) = (*l×r*)/(*n×r*) = *l*/*n*, resulting in $SCI\approx syllables\times \sqrt{unit.count^{2}-syllables\times {\left(E(m)-1\right)}^{2}}$ (see SI).

### Additional data

Species level data, for hypotheses testing, were obtained from various sources depending on the type and availability. The monogamy variable was formulated by denoting primate species that exhibited strict monogamy or social monogamy as determined by a single study [101]. Group size data was largely extracted from a single article [102] while habitat variables were obtained from secondary sources [103, 104]. Coding of arboreality erred on the side of denoting only *predominantly* arboreal species as such because nearly all primates do exhibit some degree of arboreality [105]. Territoriality was largely indicated by scent-marking behavior or daily territorial calling. Solitary species included two truly solitary species (*Nycticebus coucang* and *Tarsius srychta*) as well as several exhibiting “neighborhood” level sociality [103]. All additional data are bundled in the online score archive [106].

### Principal components and dimension reduction analysis

We used the R [107] implementation of PCA as a guide in reducing the structural acoustic feature scores from six to just three variables that could then be combined into a univariate index of musical behavior. In this dataset, for example, repetition and rhythm are highly correlated with each other (*ρ* = 0.82; *n* = 832; Spearman) as are tone and interval (*ρ* = 0.47; *n* = 832; Spearman). These two variable pairs are therefore strong candidates for reduction where one variable from each pair is kept as a proxy for both variables in the pair. The end goal of this reduction was to both eliminate redundancy and for creating a univariate outcome variable for statistical analysis. Using PCA to inform a dimensionality reduction also had several additional advantages ranging from alleviating visualization issues to addressing multicollinearity of variables [108].

**Fig 3:**
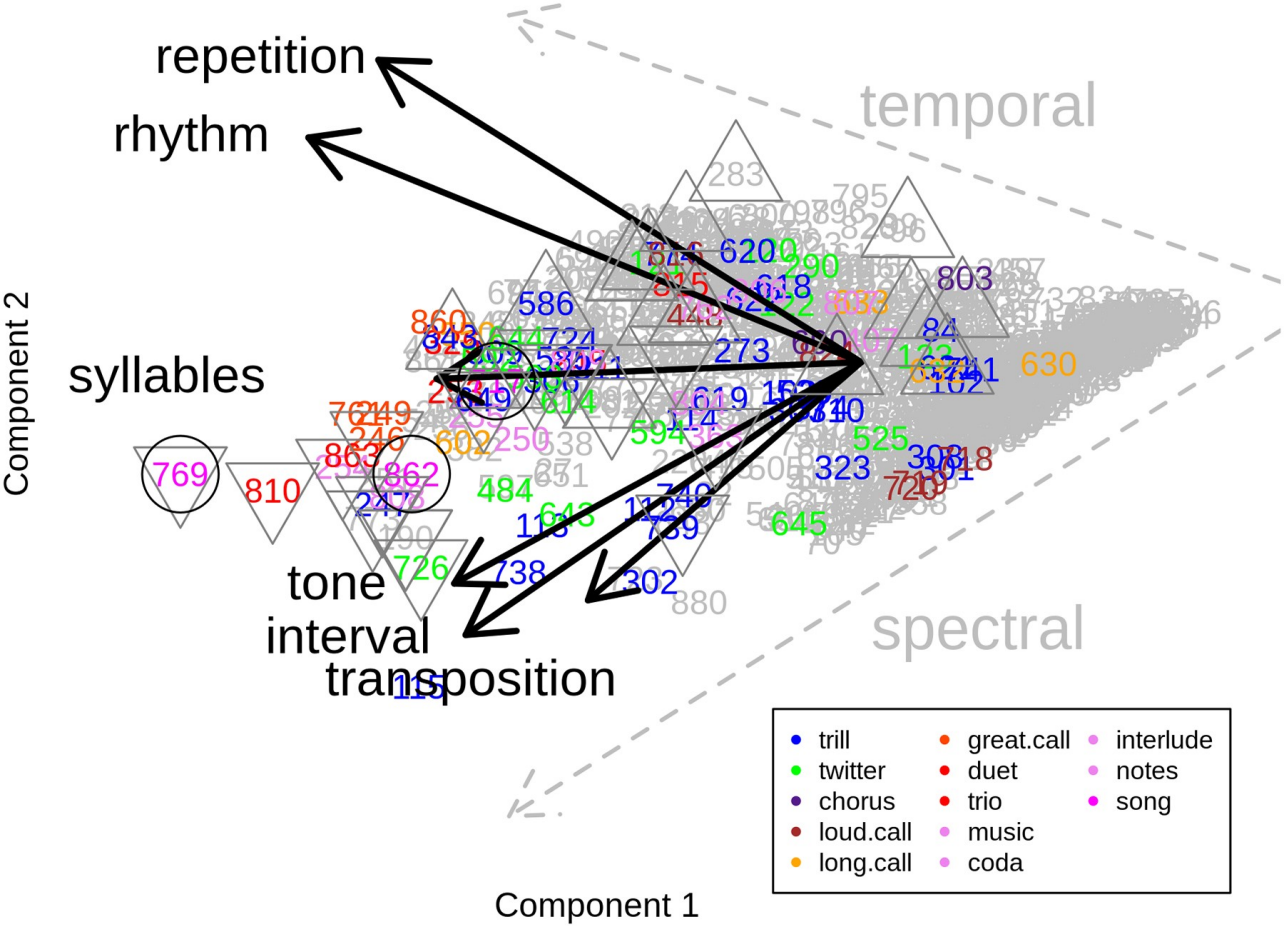
Principal components analysis [PCA] on six acoustic music universals (tone, interval, rhythm, repetition, transposition, and syllable count) where each numbered point above represents one of 832 unique primate vocalizations. Primary study author determined call types show affiliations amongst color-coded call number labels of music-like calls—as distributed across the primary dimensions (first two principal components [PC]) of this multivariate analysis. PC1 suggests that the most musical calls (far left) are distinctly more signal-rich than the long calls, loud calls, or choruses (right). Circled numbers represent the three example calls named as “songs.” The large group of gray numbers, just above and parallel to “spectral” (lower right), are all single unit calls. A second, less populated, region of non-musical calls, just below and parallel to “temporal” (upper right), reveals more periodic and repetitive calls. Each of the six arrow-head coordinates represents the loadings (or correlative contributions) of each of these structural acoustic feature scores along PC1 and PC2 (also see Fig 4). The three distinct clusters indicated by these PC loading coordinates (black arrow-heads), suggest a possible reduction in dimensionality down to just three proxy measures–a diversity measure: syllable count (left) and two redundancy measures: temporal (top) and spectral (bottom). This importance of syllable count and temporal or frequency redundancy is echoed by the avian song [69] and human music universals [6, 86] literature respectively. Downward pointing triangles around call numbers represent vocalizations with a high ARDI to SCI ratio, indicative of a more transpositional musicality (see Fig 1). Upward pointing triangles represent vocalizations with low ratios, indicating long and repetitive calls.

PCA [109] is an exploratory statistical procedure that orthogonally transforms a dataset (of *n* observations on *p* possibly correlated variables) into a set of linearly uncorrelated principal components [108]. In this case, *p* corresponds to six music universal feature scores and *n* equals 829 primate vocalizations (Fig 3). The loadings (i.e. correlations, or weights) of the original *p* = 6 variables with each of the components, are a useful way to systematically translate between the original variables and these main variance-explaining best-fit lines.

**Fig 4:**
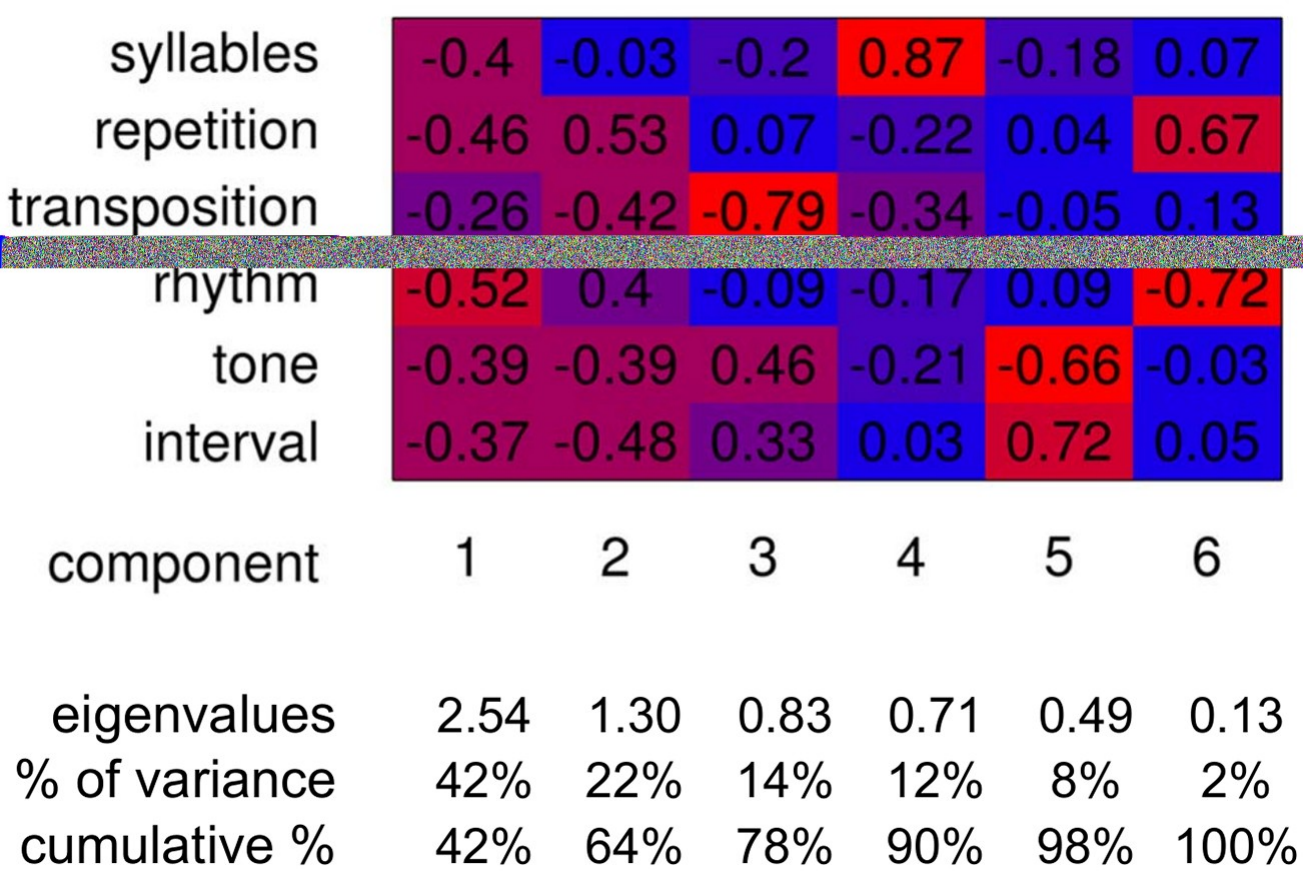
Results of the principal components analysis of music universals on primate calls. The PCA of structural acoustic features (*p* = 6) applied to primate calls (*n* = 826), suggests that repetition, transposition, and syllable count are the most distinctly explanatory of the overall variance. The feature score loadings (top) table contains each features’ correlations with each component. Red cells have high absolute loading values and blue cells are closer to zero. The variables (e.g. rhythm, tone) that are associated strongly with each rejected (λ < 0.71) component (PC5 and PC6) were eliminated. While PC1 (signal content) is relatively homogeneous across features, the retained PC2 (degree of temporal versus spectral redundancy: 22% of total var.) distinctly partitions the six factors into three groups (see Fig 3).

The loadings were used as a guide in selecting a subset of variables that encapsulate most of the variation. This involved selecting the variables with the highest loading (α_0_), or contribution, in the retained components (α_0_ > 90%) and discarding those variables associated with low eigenvalue (λ_0_ < 0.7) components [110].

For the reduction procedure, we followed the non-clustering *method B2* outlined by Jolliffe (pg. 161–162) that works from the smallest to next largest eigenvalue components (our 6^th^, 5^th^, etc.) and eliminates each variable that has the largest loading in each component’s eigenvector (a column-wise maximum in the loading matrix) [110]. We discontinued this backward-working elimination procedure once we reached components with eigenvalues over 0.71 (pg. 170) [110].

**Fig 5:**
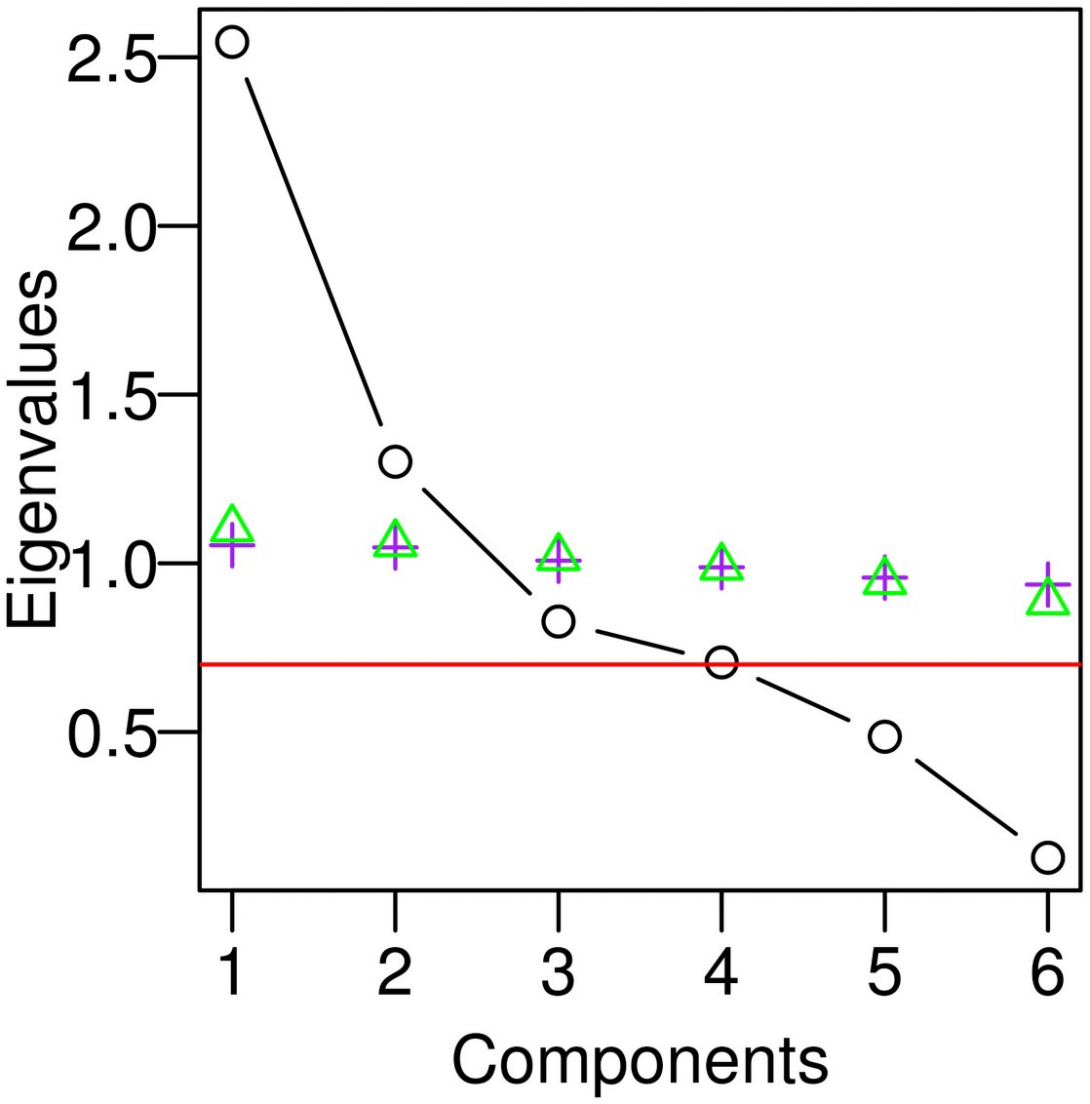
PCA variable selection scree plot with various cut-off lines. Parallel analysis, which generated a cut-off line based on a randomly simulated, similarly sized dataset (purple plus marks and green triangles), suggests retention of the first two components (left); Jolliffe’s cut-off (λ < 0.71), determined via simulation on artificial data (red line), suggests a rejection of the last two components (right). The “elbow” of this plot lies between these horizontal cut-off lines, arguably at component three. The most important musical features suggested by these various procedures prominently include repetition, transposition, and syllable count.

For the component selection procedure, additional methods were applied to the PCA results in order to confirm how many factors should be focused upon for subsequent index development. More traditional tests included inspection of scree [111] and LEV [112] plots for a pronounced inflection or “elbow” in eigenvalues as well as applying the Kaiser (KG) Rule [113] which simply divides the scree plot into components above and below an eigenvalue of one (Fig 5). We also used more recent methods including parallel analysis [114] which effectively simulates an analogous cutoff line based on *random* input values (Fig 5).

### Index development, verification, and demonstration

To develop an index that most efficiently captures acoustic display patterning in musical calls, we morphed the results of the PCA into an even smaller number of variables using a probability argument. We also used theoretical arguments—invoking norms from avian bioacoustic research, human music history, and ethnomusicological works [34, 56, 79]—to bolster this acoustic feature selection and consolidation. For verification we performed Mann-Whitney U tests and Pearson’s rank of the index against original call names and contexts. Further verification was performed to assess the redundancy and tone of putative musical primate calls in order to quantitatively distinguish them from (non-musical) human speech or other simple periodic sounds. We also illustrated the utility of the resulting index by examining theories of song and music evolution, explicitly testing monogamy and (intervalic) AAH. Preliminary tests here do not use phylogenetic control, but our forthcoming investigations, which produce nearly identical results, do incorporate these methods.

## Results and discussion

### Intercoder correlation, scoring reliability, and score replicability

Scores were reliable (Table 1) and fairly similar across individual observers (average(σ) < 2 of 10) with syllable count and transposition having the lowest deviations (σ < 0.6). Correlations [115] between the scores of coders were all high and significant (*p* < 0.001) ranging from just under ρ = 0.5 (for tone and syllable) up to ρ = 0.7 for repetition and rhythm). The scoring results had high reliability scores according to Chronbach’s alpha measure [116] ranging from an “acceptable” (α > 0.7), for transposition, to “good” (α > 0.8), for most scores, to “excellent” (α > 0.9), for rhythm (Table 1). Further validation for using the average of these scores lies in the fact that higher-order aggregation of them was even more consistent than individual scores alone (see S2 Fig). The pronounced reduction in variance from averaging scores suggests that the likelihood of replicating the means of these scores should be high. Known false positives for many of these features, however, manifest in the scores for the two-phase groan (#510) of *Perodicticus potto*, the purr (#656) of *Saimiri sciureus*, and the soft growl (#540) of *Pithecia pithecia*.

**Table 1:** Reliability and agreement between five raters for the six features of musical utterances.

syllables	repetition	transposition	rhythm	tone	interval	statistical test
0.491	0.670	0.314	0.761	0.487	0.514	Spearman’s correlation
0.826	0.895	0.716	0.933	0.821	0.833	Cronbach’s alpha

### PCA results

We performed a two-fold approach of both component reduction and component selection in deciding which components to continue focus on, in selecting our subset of universal music acoustic feature variables. For component *reduction*, Jolliffe’s (λ_0_ < 0.7) cut-off suggested rejection of the last two components (Fig 4). In component *selection*, scree plot, parallel analysis, and the Kaiser rule (Table 2) suggested selection of just the first two components. We investigated which variables loaded the highest upon each of these components (per column of Fig 4) in order to determine which features to dismiss and which to retain for further index formulation. While PCA can also be employed to automatically capture systematic variation due to latent variables, such as cognitive musical-pattern processing predispositions, we do not specifically test for such latent factors here.

**Table 2:** Retained PCA components and stopping rules. All rules suggest retaining at least the first two and perhaps as many as four components. The last two components (that distinguish tone/interval and rhythm/repetition) were discarded based on λ < 0.71. PC2, the most reliably informative component, spreads the six variables into three distinct clusters (Fig 3) and is retained under all stopping scenarios.

rule	λ	% CV	comps.	display	source
Cumulative Var.		90	4	Table 4	
Scree Test			3	Fig. 3	Cattell, 1966
Log scree / LEV			3	Fig. 3	Farmer, 1971
Kaiser-Guttman	0.9		2	Table 4	Kaiser, 1960
Parallel Analysis	1.0		2	Fig. 3	Franklin, 1995
Jolliffe’s KG	0.7		4	Fig. 3	Jolliffe, 1972
Broken Stick	~1		2	Fig. 3	Cangelosi, 2007
Bartlett’s Test			3	Fig. S1	Bartlett, 1937
Velicer’s MAP			2	Fig. S1	Velicer, 1976
Revelle’s VSS			2	Fig. S1	Revelle, 1976

The results of the PCA (Fig 4) suggest that PC1 (the best-fitting variance-minimizing line) delineates along a continuum from the musical—signal-rich, song-like and “musical” calls—to the palpably non-musical—those that are acoustically noisy, prosaically periodic, or simple single unit calls (Fig 2). We hereafter refer to this as the “signal content” component. All loadings in this component are in the same direction (negative) suggesting that all six features contribute to explaining the signal content component and are helpful in assessing the structural acoustics of musicality. A broad and unanimously oriented dispersion along this musical signal-pertinent component (substantiated by all of our six select musical features) heretofore provides solid quantitative evidence supporting the notion that many primate calls could be considered as having musical qualities (Fig 3: PC1). This first signal content component explains 43% of the variance (Fig 4) but is relatively homogenous in factor loadings across the structural acoustic features and therefore may not be as informative for distinguishing calls, or for variable reduction, as other components.

The second component, which minimizes the variance between the first component and the residuals of that component’s fit, differentiates between types of redundancy: temporal versus spectral (Fig 3 top and bottom respectively). The highly correlated time-domain measures of rhythm and repetition both have positive loadings and the spectral domain measures of tone, interval, and transposition all have negative loadings along PC2. This single component is perhaps the most unambiguously meaningful for our purposes of informing a variable reduction of the six features to just three—not only due to its heterogeneity in loadings and high explanation of variance (22%) but because it draws attention to different types of redundancy, a key [117] and oft-neglected feature of acoustic musicality [85, 86].

A pronounced inflection point in eigenvalues between these first two components (PC1: signal content: λ = 2.56 and PC2: redundancy: λ = 1.3) and the rest suggests that we might focus primarily on the former and less on the latter. The third and fourth components, however, do explain a good proportion of the overall variance—raising it 25.5% from 64% to 90%—and the eigenvalues (λ_0_) are all above 0.7 and suggest retention [110]. These two components are harder to interpret than the first two (signal content and redundancy type), but the loadings correlations, of each parameter with each component, are informative. The single highest loading for each of these two components is, interestingly, transposition (79% loading correlation) and syllable (87% loading correlation). They explain 13.8% (PC3) and 11.7% (PC4) respectively of overall variance—after PC1 (42.5%) and PC2 (21.6%).

Syllable count is the most unambiguously neutral in PC2 (redundancy) and clearly collimate with PC1 (signal content) suggesting it could be an efficient indicator of complex calls. As mentioned above, it was also the highest loading feature in the 4^th^ component—one which explains 12% of the variance of the overall dataset. Syllable diversity’s prominence is not that surprising as its analog (repertoire size) is a commonly used metric for display quality in avian acoustic research [69, 72, 74]. Our focus on syllable here is nicely supported by research highlighting songbirds use spectral shape for sound pattern recognition [118].

Repetition and rhythm had similar loadings in PC1 and PC2 (Fig 3) suggesting a collapsing of them into a single variable to reduce collinearity. Rhythm was indicated as being important, but it was excluded from the index due to its high association (72%) with discarded PC6 (λ = 0.13). Only one of these two features was retained as either one could serve as a rough proxy for time-domain redundancy. Repetition is more elemental (as it is often a *prerequisite* of rhythm) and is thus considered to be further justified for retention in the index. This decision to preclude rhythm is echoed by beat entrainment research disputing Darwin’s assertions on rhythmic abilities (especially anticipation) in animals [119]. We offer additional rationale below in arguing for rhythm’s more appropriate classification as a musical *system-level* universal (also see Fig 2).

The PC1 and PC2 loadings for tone, interval, and transposition similarly overlap with each other in the PCA analysis (Fig 3 bottom) and could be reduced to a single representative non-co-linear variable representing frequency domain redundancy. Emotive/arousal universals, the un-assessed variation in tempo and amplitude, as well as our measure for interval, were difficult to properly gauge. And as it had the highest loading with the discarded fifth component (λ = 0.49), interval was precluded. Pitch, like rhythm, has an unclear position in the gradient of musical universality somewhere between utterance and system-level universals [34, 56], and it is possible that tonal (pitched) units should not be categorically required in an utterance level definition (Fig 2). This finding is corroborated by work on universality of dissonance-based scale structure [120], and work finding that songbirds use spectral cues other than absolute pitch for pattern perception [118]. Transposition, with its high loading on the third component, was selected to serve as a proxy for both pitch and interval.

### Towards a univariate quantitative index

Music has simply and broadly been encapsulated as an emergent balancing of ritualization with innovation, or “an unusual combination of order and chaos” [13], of “redundancy balanced by variety” [34], of versatility with continuity / monotony [121, 122] of expectation with surprise [123], or “internal repetition with variation” [56]. Variety unquestioningly provides the combinatoric uniqueness underlying musical novelty and interest. But its counterpart, repetition, though it serves as foundational temporal acoustic scaffolding for constructing human musical displays [86], remains an analytically neglected [85] and relatively unsolved puzzle for researchers in evolutionary musicology. As mentioned in the PCA results above, our analysis provides compelling evidence that features prevalent in human music also appear, to varying degrees, in primate calls (Fig 3: PC1). These qualitative observations on organizational balance, however, suggest that our essential musical features *in combination* with each other could create something unmistakably, if not uniquely, musical—even though the features *in isolation* may not quite resemble the larger emergent whole.

Thus, finding ways to combine these elements should help us find a suitable formulation of manifest musicality. We need only include this minimum set of structural acoustic universals, as we are most interested in detecting musical utterances at the most abstract, general levels. And we could require the two simplest, yet neatly and contrastingly balancing, features of redundancy (sometimes measured as *consistency*) and variation (often proxied by *size* or *complexity*) of units (e.g. syllables or melismatic phrases) within an utterance—especially given the quality metric overlap (avian and human) discussed in the Introduction. Furthermore, the PCA conveniently corroborates this theoretical argument for a simple inclusion of just these few non-collinear variables.

We still need to quantitatively combine this minimal set, however, if we are to obtain a single outcome measure of elaborate structural acoustics. While the PCA indicates focusing on three variables, the more intuitive arguments, in the paragraphs immediately above, compel us to focus on only two: the contrasting features of sameness and difference. Syllable diversity is an obvious choice to represent difference or *variation* while repetition and transposition are reasonable choices to represent forms of sameness or *redundancy*. Hereafter we provide the mathematical rationale for adding the two redundancy measures together first and then multiplying the result by our *syllable* variation measure.

These two (within-utterance) features can be quantitatively defined as follows: *variation as* a count of the number of distinct syllables and *redundancy* as reappearance of syllables across time—either at the same frequency, in the case of repetition, or at different frequencies, in the case of transposition. Mathematically, we need to determine which operations to use when combining these together. As for combining repetition and transposition, we can re-purpose the addition rule of probability theory [124] that states that for two events, A and B:

P(AorB)=P(A)+P(B)−P(AandB) (1a)

The last term can be set to zero due to mutually exclusivity [124] of the repetition and transposition of any given vocal unit. That is, it’s impossible to both repeat, in time, and transpose, in frequency, a unit across an entire call. These redundancy features capture highly similar, albeit dimensionally orthogonal, acoustic phenomena—differing in that one measures it in time the other in frequency. Since the two feature scores also happen to be easy to scale (after dividing by 10) into probabilities, as they are already recorded on a scale of 1 to 10, the probability of unit reappearance, as the sum of the two terms, can be written as:

P(reappearance)=P(repetition)+P(transposition)−0 (1b)

It is important here to note that *reappearance*, when calculated using human-observation-based scores instead of computational unit-clustering methods, may not reliably yield true probabilities—since the joint probability term is only approximately zero—and therefore may need to be rescaled or otherwise re-bounded between zero and one.

For integrating this new reappearance probability into our index, we can model the index (which requires both unit reappearance and syllabic diversity) as an expectation [124] written like so:

$$\text{E}(X)=\sum \left(x_{i}\times \text{P}\left(X=x_{i}\right)\right)$$ (2a)

where X is a random variable that serves as an indicator of reappearance. It is a binary (yes or no) variable that answers the question: does this unique syllable [i] occur elsewhere in the utterance? The probability term can be removed from the summation because it is uniform across the entire call (scoring was assessed on entire calls and not individual units). The equation, within the context of this study, then simply becomes the count of unique syllables times the overall probability of syllable recurrence within the utterance:

$$\text{E}(X)=N\times \text{P}(X)=\sum \left(x_{i}\right)\times \text{P}\left(X=x_{i}\right)$$ (2b)

Rewritten with the full names of the two main components, this expectation looks like:

E(numberofsyllablesreappearing)=syllablecount×P(reappearance) (2c)

This use of multiplication is an elegant and mathematically certain way to require that each of these elements co-exist within every musical utterance; *multiplication* of the two individual feature scores of syllable and reappearance guarantees a score of zero if either feature is scored as zero (Eq 2c). Likewise, low syllabic diversity or low reappearance will necessarily result in a low ARDI score. Armed with a reconsolidation of musical feature components into this univariate index, we can now more easily and fruitfully perform statistical analyses and visualizations to help independently understand the evolutionary origins of such musically endowed calls.

### Corroboration of the index

**Fig 6:**
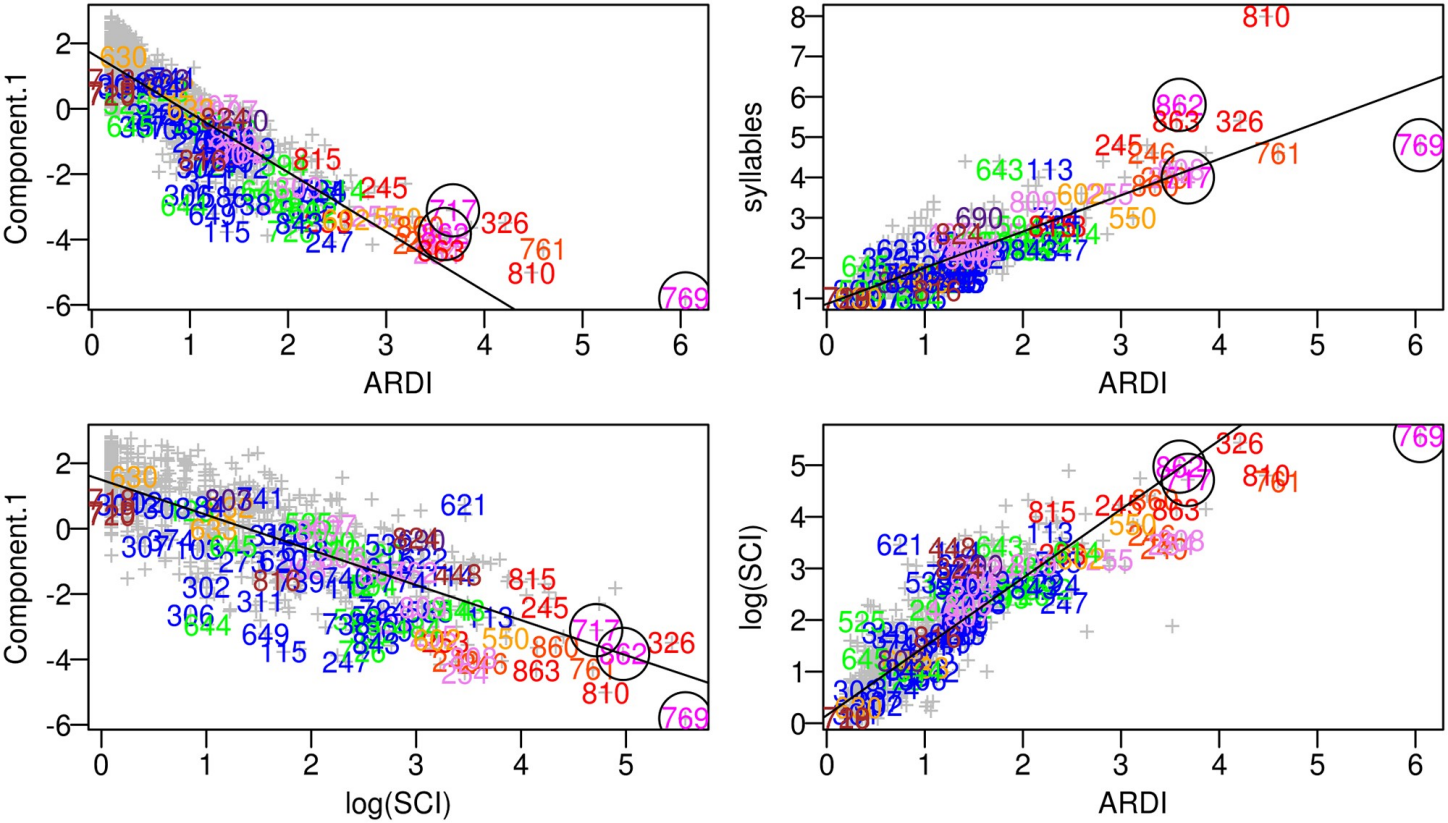
Scatter plots of ARDI versus various other possible metrics of acoustic musicality. Colors are coded the same as in Fig 3 (with warmer colors corresponding to more human music relevant names) and circles enclose each of the three primate songs: *Indri indri*’s: “song” (#717), *Tarsius spectrum*’s “duet song” (#769), and *Hylobates leucogenys*’ “trio song” (#862). ARDI identifies fewer false positives* than both PC1 (e.g. trills and twitters) and syllable count (e.g. #643 twitter & #113 trill) whereas log(SCI) captured songs more efficiently*. SCI tends to reward longer, more repetitive calls such as the loud call (#448) of *Macaca silenus*, the duet of *Lepilemur edwardsi* (#815), the chirrup pumping (#46) of *Callicebus moloch*, or the pant-hoot-drum (#820) of *Pan troglodytes*. ARDI, alternatively, tends to boost scores of shorter, more transpositionally musical calls such as the gothic chucks (#648) of *Saimiri sciureus*, u-trills (#113) of *Cebus olivaceus*, the twitter (#726) of *Tarsius syrichta*, or the trill (#247) of *Hylobates lar* (see also Fig 1). Note that SCI is arbitrarily scaled, but conveniently happens to align well with the scales that manifest for syllable count and ARDI. (*compared with researcher designations).

**Fig 7:**
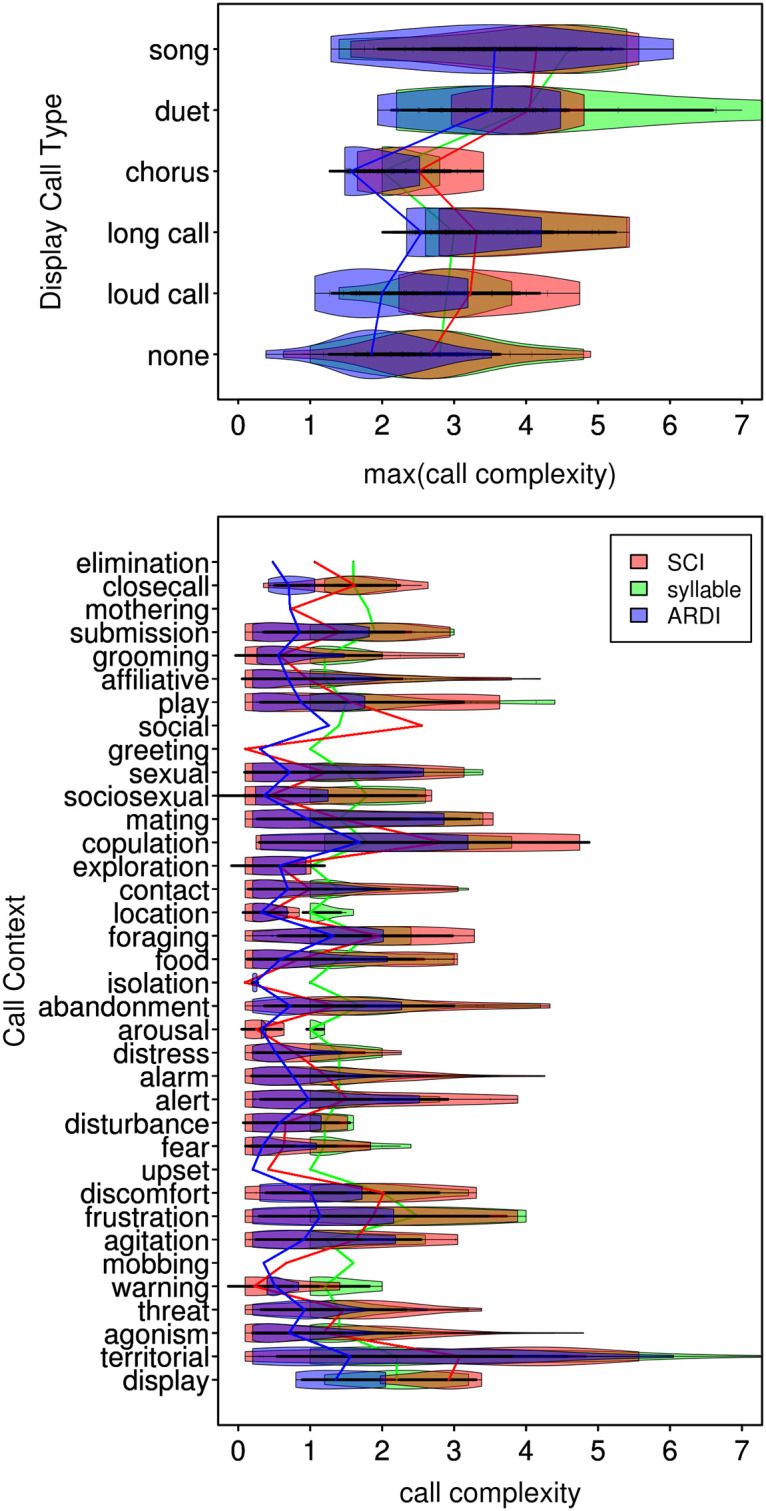
Corroborative violin plots of complexity scores versus call types and contexts. **a.** High correspondence with “duet” and “song” is seen for all complexity metrics. SCI, which favors length over other features, also captured longer calls. *Chorus*, interestingly did not correlate highly under any metric. ARDI scores were relatively lower for calls in the non-display types than SCI and syllable, potentially reflecting a robust specificity. **b.** Outwardly focused social, display, and territorial calls are the primary vocalization context that appear to strongly associate with higher call complexity values. The relatively higher ARDI scores for foraging and exploration contexts provide support for identity and location-based signaling [126] as part of AAH. The lower scoring sociosexual (versus actual copulation) contexts lend less support for evolution by sexual courtship mechanisms of musical evolution. ARDI most strongly rivals SCI in several contexts—such as grooming, mothering, isolation, greeting, location, and exploration—suggesting that primate musicality could have originated in familial foraging or parentally chaperoned settings. Color overlaps produce additive mixes between rgb colors on the color wheel (e.g. amber = green+red, violet = blue+red).

We illustrate the verity of the *acoustic reappearance diversity index* ARDI by demonstrating its efficient capture of musical names (Table 3 and Fig 6) as well as its correlation with vocalization categories and contexts as designated by primary researchers. The appropriateness of the composite index was suggested by its assignment of relatively higher values to vocalizations described as *song*, *duet*, *trio*, *chorus*, *great*, *music*, *scale*, *coda*, *intro*, or *interlude* (Pearson’s rank, *n* = 829, *r* = 0.49). Visual evidence of these correlations is available by inspecting the overlay of these song names on the PCA plot (Fig 3). The correlation between higher *acoustic reappearance diversity index* values with classifications such as *duet* or *song* (Wilcox-test: *n* = 58, *W* = 91, *p*<0.01; Fig 7a) verified this composition of features in the composite score [125]. These scores are univariate, continuous, blindly scored, and also conform to expert-determined names and contexts.

**Table 3:** The ranking of all primate songs and duets by four acoustic complexity indexes.

	genus	species	id	name	ARDI		syllables		Comp. 1		log(SCI)	
**songs**	Tarsius	spectrum	769	Duet.Song	6.0	1	4.8	6	-5.8	1	5.6	1
	Indri	indri	717	Song	3.7	6	4.0	21	-3.1	35	4.7	7
	Hylobates	leucogenys	862	Trio.Song	3.6	8	5.8	2	-3.8	13	5.0	3
**duets**	Tarsius	spectrum	769	Duet.Song	6.0	1	4.8	6	-5.8	1	5.6	1
	Hylobates	pileatus	810	Great.Call.Duet	4.5	3	8.0	1	-5.0	2	4.8	5
	Leontopithecus	rosalia	326	Long.Call.Duet	4.2	4	5.4	4	-3.5	23	5.4	2
	Hylobates	concolor	863	Great.Call.Duet	3.6	9	5.4	4	-4.3	4	4.1	16
	Hylobates	agilis	245	Great.Call.Duet	3.0	20	4.8	6	-2.4	76	4.2	13
	Hylobates	lar	253	Duet.Intro	2.4	36	2.8	74	-3.5	25	3.3	59
	Lepilemur	edwardsi	815	Duet	2.3	45	2.8	74	-1.5	154	4.1	17
**musical**	Hylobates	pileatus	808	Wow.Waoo.Notes	3.6	7	4.2	17	-4.0	11	3.5	33
	Hylobates	lar	254	Quaver.Interlude	3.5	11	4.0	21	-4.4	3	3.5	43
	Hylobates	pileatus	809	Waoo.Notes	2.1	61	3.4	35	-2.4	75	3.1	79
	Hylobates	syndactylus	762	Interlude.Sequence	1.5	147	2.2	166	-1.3	187	3.0	93
	Hylobates	pileatus	804	Wa.Notes	1.5	158	2.2	166	-1.4	179	2.7	122
	Hylobates	pileatus	807	Oo.Notes	1.5	170	2.2	166	-0.1	358	2.1	224
	Hylobates	pileatus	806	Leaning.Wa.Notes	1.4	173	2.0	219	-0.9	245	2.3	182
	Macaca	fuscata	363	Musical.Sequence	1.3	208	2.2	166	-1.2	193	1.9	250
	Macaca	nemestrina	407	Intention.Notes	1.2	228	2.6	96	0.1	382	2.2	210

All three primate songs (**top**), all eight primate duets (**middle**), and all nine musical calls (**bottom**) ordered by decreasing ARDI scores, with each rank on right, demonstrate its high efficiency in capturing these types of calls as designated so by western researchers, when compared with other reasonable alternatives such as syllable count, SCI, and the first component (PC1). This is noticeable with respect to *Indri*’s (relatively more repetitive) song: only ARDI and SCI effectively capture it in their top ten lists of musical vocalizations (syllable: 21st and Component.1:35th). ARDI, like Component 1, also bears an advantage of a high true-positive rate for identifying researcher-determined musical calls. Both Component 1 and ARDI identified eleven calls with musical elements (e.g. “long call” and “aouuo”) as the highest scoring compared with only nine for syllable (e.g. “frustration screams” and “roar” instead respectively). Using the first PCA component alone, however, tends to overvalue tone and undervalue repetition and syllabic diversity—as evidenced by the high rank numbers for the noisier duets of *Leontopithecus* (23^rd^) and *Lepilemur* (154^th^) shown in the third column above. SCI performed better than ARDI at confirming western researchers’ designations of song. But ARDI, on the other hand, greatly outperforms SCI in identifying what western researchers deem “musical” or having “notes” as evidenced by ARDI out-ranking SCI in two-thirds of these (bottom table). Furthermore, unlike the first PCA component and SCI, ARDI is theoretically more relevant to humans, incorporates multiple explanatory factors, and is easy to interpret. SCI, unlike ARDI, is arbitrarily scaled.

In addition to capturing the musical, ARDI also does an adequate job of obviating the non-musical. ARDI successfully assigned low scores to non-musical utterances—especially single unit and simpler periodic calls (Fig 3: right). Although noisy calls were not explicitly ruled out, they tended to have lower *tone* scores (less than 0.5) by association with low syllable and reappearance assessments—likely due to low unit differentiability. *Rhythm* was likewise lower (less than 0.3) for associated low ARDI scores, even though ARDI does not directly incorporate *rhythm*. Human music is composed of sounds that are typically circumscribed within perceptual bands [64]—psychoacoustic limits and preferences for perceiving frequency, loudness, roughness, and periodicity of soundwaves [127]. But there are interesting abiotic (e.g. mechanical or environmental) sound patterns [128] as well as zoological sounds (e.g. ultrasonic animal calls) that ARDI can capture, as it is not necessarily limited by human biology. Thus ARDI allows for exploration of non-human “musical” sound patterns by transcending the quantitative limitations of human perceptual constraints, while still maintaining a high level of fidelity towards qualitatively fundamental human aesthetic universals.

Furthermore, we also distinguish between how our ARDI determined musical (primate) vocalizations differ from complex (human) speech sounds. Complex acoustic utterances encompass both complex pre-human speech as well as human-like musicality. Indeed, both language and music likely derive from a common origin [62], and share many structural similarities, such as prosodic or melodic contours respectively, especially in infant interactions [129]. But dissimilarities also abound, as musical vocalizations are far more tonal and redundant than speech. For example, the vast majority of human music is tonal and tends to be highly redundant, whereas only a handful of the world languages are tonal and most are estimated to contain only 30% sonorants [130]. Furthermore, human languages have evolved to be more efficient by eliminating unnecessary redundancy—the redundancy ratio, even for just consonants, for example, is estimated to be quite low [131]. In non-human primates, among our putative musical calls, (ARDI > 2), the average *tone* probability was 0.67 and the *reappearance* probabilities averaged even higher at 0.82 (both with overall means near 0.51). These numbers corroborate such a high level of tonality and redundancy in our more musical primate calls.

Our validation checks seem to corroborate our index formulation, but there admittedly still exist many potential cultural biases influencing both our scoring and our validation processes. The primary data collectors, scorers, and trainers are culturally western and English speaking. Additionally, the feature definitions and universals themselves were determined by western authors. Put another way, research such as this is admittedly biased because it was conceived and performed in a W.E.I.R.D (western, educated, industrialized, rich, and democratic) country and thus substantially modulated by its cultural history [132, 133]. Thus there remains some circularity in validating an index built upon western feature definitions, scored by mostly western students, and using western researcher-determined call names. Furthermore, it should be noted that after the inception of this project, more empirical studies have been published which have shifted ideas of music universals to include new features such as motivic and arousal factors [46, 56] that were not specifically accounted for in our data collection design. It is additionally important to point out that many of the features of music we investigate here have since been proven to be not nearly as universal as they were once thought [49]. Many of the scale-based features such as pitch, interval, and tone have been called into question [134, 135] as being more culturally influenced [46, 136] than biologically determined.

Our formulation was explicitly intended, however, to be independent of culture, context, mechanism of learning, mode of generation, path of evolution, and taxonomic position. Although we were not able to completely avoid all forms of cultural bias or definitional circularity, we have earnestly attempted to minimize these influences. It seems unlikely that such cultural biases would have a significant impact on the results as we strove to be as broad, objective, blind, and all-encompassing as possible. Most notably, because our scoring session relied on *visual* information to assess spectrographic data, it circumvents most *auditory* biases. Further, the most culturally sensitive features—pitch, interval, and tone (see the previous paragraph)—did not get selected for integration into ARDI anyway. With respect to scorer training and analysis confirmation bias concerns, we are motivated to avoid any context-burdened definitions as they ultimately impede interspecies comparison and hinder us in progressing beyond outdated notions of the biological separateness of humans from animals. Indeed, no study of inter-species evolution of musicality is possible without also grappling with the bias inherent to studying ourselves.

Our bias in procuring musical features as part of the PCA is admittedly, yet unavoidably, anthropocentric since one goal is to understand evolutionary genitors of *human* music. Nonetheless, the resulting composite metric may also help to *overcome* subsequent bias in evolutionary analyses. ARDI is a continuous construct that best enables consideration that human musicality evolved gradually from the calls of our primate progenitors. Our study does not find tonal and rhythmic features to be as useful in differentiating primate calls from one another (Fig 3), though perhaps this is primarily due to the relative rarity of said features. Yet to disqualify primates as amusical based on the rarity of rhythm and tonality in their calls, or perhaps due to the incompleteness of our metric, would be overhasty. Nevertheless, calls of primate species previously considered to be musical, such as indri, gibbons, tarsiers, and several genera of new world monkeys [137], all possessing calls with an ARDI score well above three, do often exhibit all of the six musical features studied here anyway (Fig 3). Therefore, despite potentially anthropomorphizing, this approach may be the best enabler in the search for a zoologically-broad evolutionary origin of musical behavior.

### Testing habitat acoustics and social effects using a species-level index

**Fig 8:**
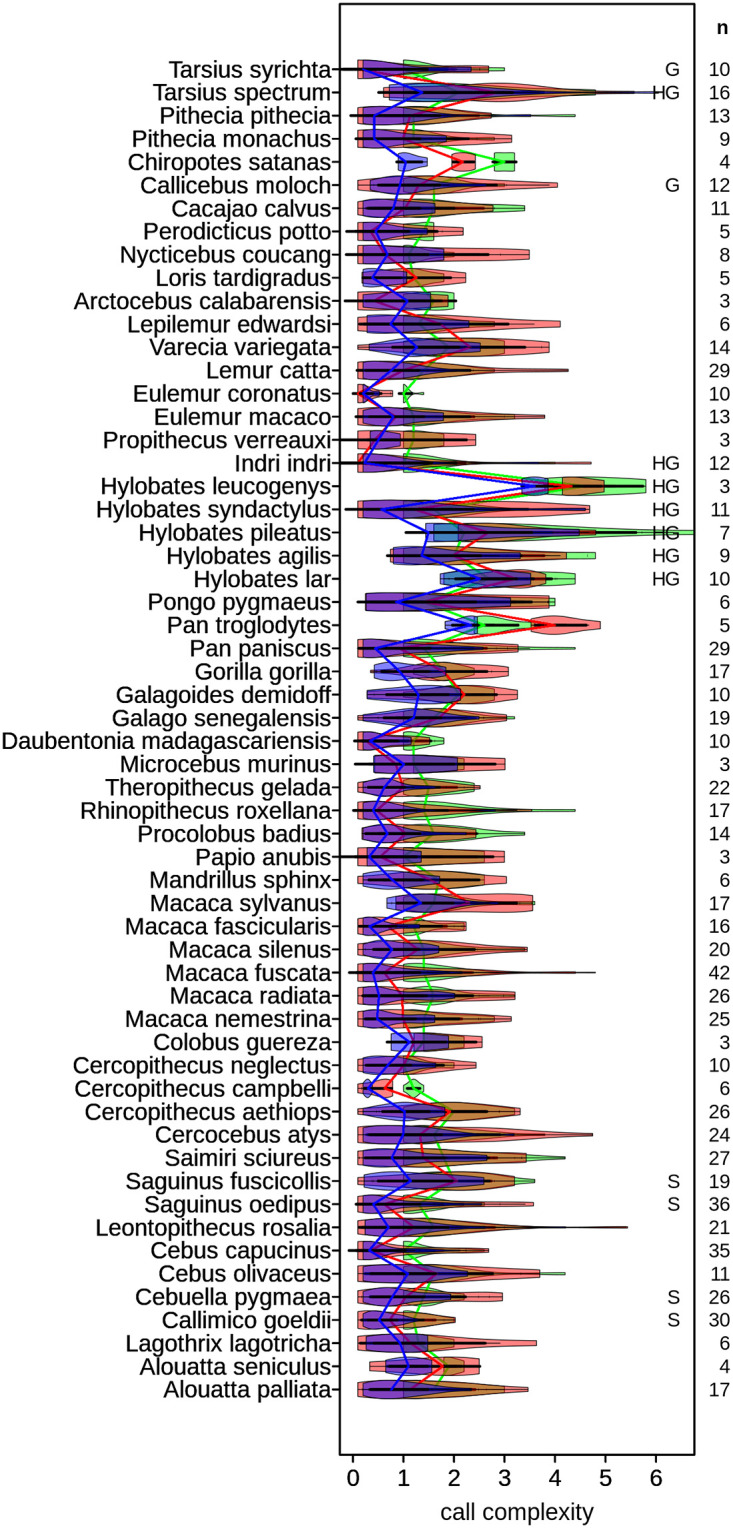
Species level call complexity score distributions for three metrics. ARDI (blue), SCI (red), and syllable count (green) complexity distributions demonstrate how species can have a great diversity of calls whose scores are quite low but have a stand-out call (e.g. *Indri*’s song) which scores exceptionally high. Total count of calls in each repertoire is listed under “**n**” on the right. Letters on the right show concordance with Geissman [137], Haimoff [39], and Snowdon [138] who previously explored these primate species for having musical capacities. Note that many new species of primate emerge has potentially possessing music-like calls including galagos, several additional species of lemur, capuchins, squirrel monkeys, multiple leaf monkeys, and even some cheek-pouch monkeys. The higher scoring distributions of the gibbons and chimps are likely due to primary researcher selection bias for recording more display-like calls. Their close / soft calls were likely left truncated out of being included in these distributions. Note that the complexity values used for evolution analyses are sampled from the far right side of these distributions. That is, we used max(ARDI) for species-level metrics (see Figs 7a and 9 also). Interestingly, ARDI scores begin to overlap with SCI in more solitary primates such as many nocturnal prosimian species. Colors are the same as in Fig 7, with overlaps producing additive mixes between rgb colors on the color wheel (e.g. amber = green+red, violet = blue+red).

**Fig 9:**
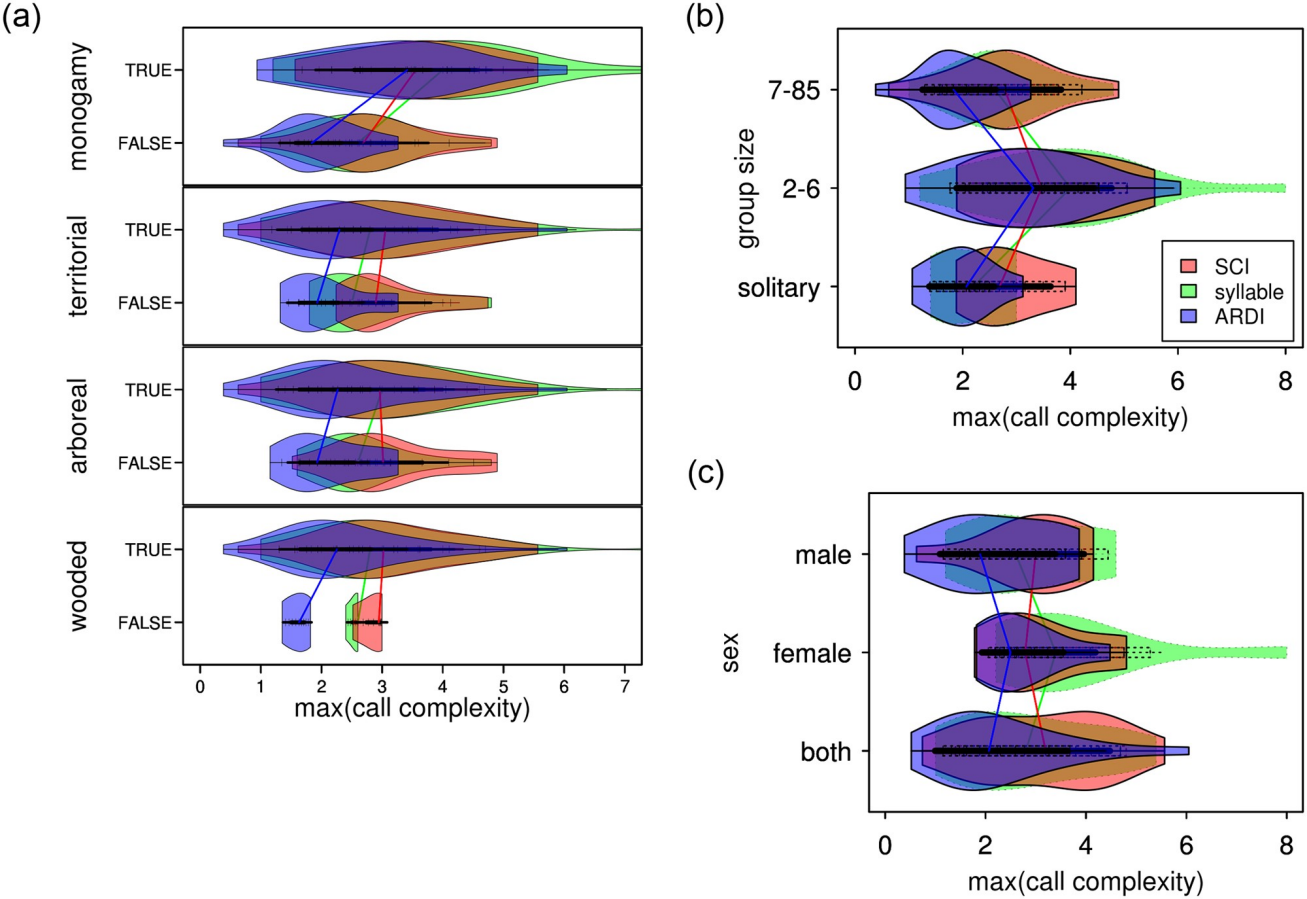
Violin plots of call complexity versus socioecological variables. **a.** Music origins hypotheses include territorial defense, pair-bonding, [139] and acoustic adaptation to habitat. The mean scores for each of these species level factors exceed their corresponding false values, indicating all of them could have an influence on display musicality. Social monogamy appears to have the strongest association with call complexity. ARDI has results that parallel syllable count with scores generally a full integer lower after discounting by its reappearance factor. SCI interestingly seems to be much less influenced by habitat factors than ARDI which has strong responses in both *wooded* and *arboreal*. But also notice that ARDI has the steepest slope differentiating *monogamous* status. Colors are the same as in Figs 5 and 7b, with overlaps producing additive mixes between rgb colors on the color wheel (e.g. amber = green+red, violet = blue+red). **b.** The highest vocal complexity scores occur in small group size species (e.g. duetting primates such as gibbons, tarsiers, and callitrichids). ARDI scores for solitary, neighborhood-living species were robust against down-weighting by *reappearance*. SCI similarly seems to be less influenced by group size factors than ARDI but confirms that smaller groups may exhibit a more noticeable musicality. **c**. In our species level dataset, the most complex calls (via ARDI and syllable) are female generated. SCI, however, seems to indicate that vocalization types performed by both sexes are more complex. Together, these results possibly contraindicate sexual selection (for male display) primarily driving call complexity in primates. Alternatively, however, females (e.g. gibbons) could instead be attracting extra-pair mates as a side-effect of their territorial (e.g. great) calls, in counter-point to social (and pair) bonding theory.

We used a single index value for each species to explore questions about musical behavioral origins. The possible scores within a species, from which the top score was selected, were notably varied (Fig 8). The maximum score for each species was used because we are ultimately interested in the highest degree of possible performance in the display calls of species. This maximum (highest score per species) ARDI formulation showed negligible correlation with many possible study and species level predictor variables, but significant exceptions such as habitat, monogamy (Fig 9a), and group size (Fig 9b) are discussed hereafter.

The first hypothesis we considered was that of acoustic adaptation (AAH), specifically the inter-element interval aspect. The hypothesis suggests that vocal animals should evolve long, low, intervallic, or otherwise propagative calls that overcome vegetative obstructions in habitats that absorb or muffle acoustic signals [14]. We were not able to test the fundamental-frequency-based component of AAH as we had focused on tabulating more relativistic musical parameters. The data presented here do, however, suggest support for the second part of the AAH regarding inter-element intervals, as ARDI’s transposition factor is accentuated by such larger, more noticeable intervals. Foraging, which often requires both distance and contact between solitary travelers, had the second-highest ARDI scores of nine higher-order call context groupings—and long calls were second behind musical and display calls implying distance relevant influences on vocal elaborateness. Furthermore, species living in forest habitats had a call with 0.75 (on average) more reappearing syllables (*t* = 3.77, *df* = 9.74, *p* = 0.004) which seems to suggest that changes in habitat acoustics could moderately promote the musical structure of calls (Fig 9a). We fail to reject the AAH here but recommend the development of more sophisticated metrics, such as ARDI, in future tests on AAH using data from other species. Additionally, richer variable types (e.g. beyond our merely binary arboreality measure) should be used to better explore the effects of higher-dimensionally structured habitats and associated behaviors.

The next body of ideas we considered concerned the effects of sociality on elaborate acoustic display behavior. Our index and dataset modestly support pair bonding [4] and group cohesion [25] but also, to a greater degree, the signaling by [11] and selection for [31] small groups as important coevolutionary factors driving the evolutionary precursors to human music. Monogamous species had 1.2 (on average) more reappearing syllables for their most-elaborate call (Fig 9a). We found less support for a strictly positive linear correlation with group size, but our analysis does indicate that species living in small-sized groups (*n* = 2 to 6) possessed more song-like calls (Fig 9b). Compared with large groups or solitary species, small groups had almost 50% more reappearing syllables (on average) in their most-elaborate call (*t* = 3.58, *df =* 20.1, *p* = 0.002). The fact that sociosexual and mating calls scored lower than many other call types (see Fig 7b) suggests that conventional sexual selection might play a less prominent role (also see Fig 9c) than other more egalitarian within-group factors in influencing the evolution of musical calling. The discovery that female calls have higher than average ARDI scores (Fig 9c), however, inspires the alternative interpretation that socially monogamous females could accentuate a (e.g. great) call not only to repel resource rivals (e.g. neighboring mated pairs) but to simultaneously solicit copulations from extra-pair males. This paradoxically opposing dual function is similar to the “outgroup exclusion” side-effect of social bonding [24], but opposite in that extra-pair bonds are developed instead of those within a mated pair. Although testing other social influences—emotional communication, language acquisition, and many large group phenomena—is out of the scope of the present work, these interrelated factors could still prove consequential, but do not stand out as significant here in primates.

While we have considered the social and habitat influences on patterned acoustic signals *separately*, we are partial to the idea of their evolutionary interdependence and modularity [6, 40, 44, 45]. Even our most sophisticated social utterances (e.g. poetic language), rest upon the *precursors* of a more basic mammalian need for acoustic information sharing [140]. The biological antecedents of this basic communication are predicated upon distinguishing an acoustic signal from background noise and augmented by appraising complex patterns over simpler periodic sounds (see PC1 in Fig 3). Thus many of these purported influences on acoustic display could manifest at different times, multi-factorially [44, 141, 142], rather than as mutually exclusive evolutionary pathways. The manifest modularity constituent to ARDI elegantly provides a missing-link for the testing of evolutionary theories—enabling simultaneous investigation into both more proximate display and signaling influences as well as more ultimate habitat-oriented selection pressures [143]. Such modularity could further enable temporal, taxonomic, and theoretical bridges between ancient and modern, between non-human and human, as well as between these habitat-driven and socially-determined forms of embodied musicality.

### Conclusion

Musical behavior is ubiquitous amongst humans but similar vocal behavior also appears to have evolved in globally dispersed animal taxa as well [58]. Our study provides quantitative evidence that many primate vocalizations contain features foundational to human music (Fig 3: PC1). Our index also efficiently detects acoustic musicality (Fig 6, Table 3) in a way that helps unify musical terminologies and could be applied broadly to other species. In research on bird song, for example, assessing *complexity* by measuring syllabic repertoire size is common [74], but it also appears to be a suitable measure of *variation* in more anthropocentric song as well. Other feature overlaps between the structure of bird song and human music exist, and in particular, various forms of *redundancy* (e.g repetition) are fruitful to study as they are often neglected [85, 144] and are not typically captured via *complexity*. For a more broad and objective characterization, we focused on the sound itself rather than contexts or mechanisms (e.g. culture, learning, production, or evolution). Additionally, by focusing on utterance level features—those applicable at the smallest durations, we were able to concentrate on the most broadly applicable universals, even encompassing birds and other animals. Borrowing theory and methods from avian bioacoustics, we looked for evidence of these human music universals (e.g. interval, repetition, tone) in our nearest cousins—the non-human primates.

**Fig 10:**
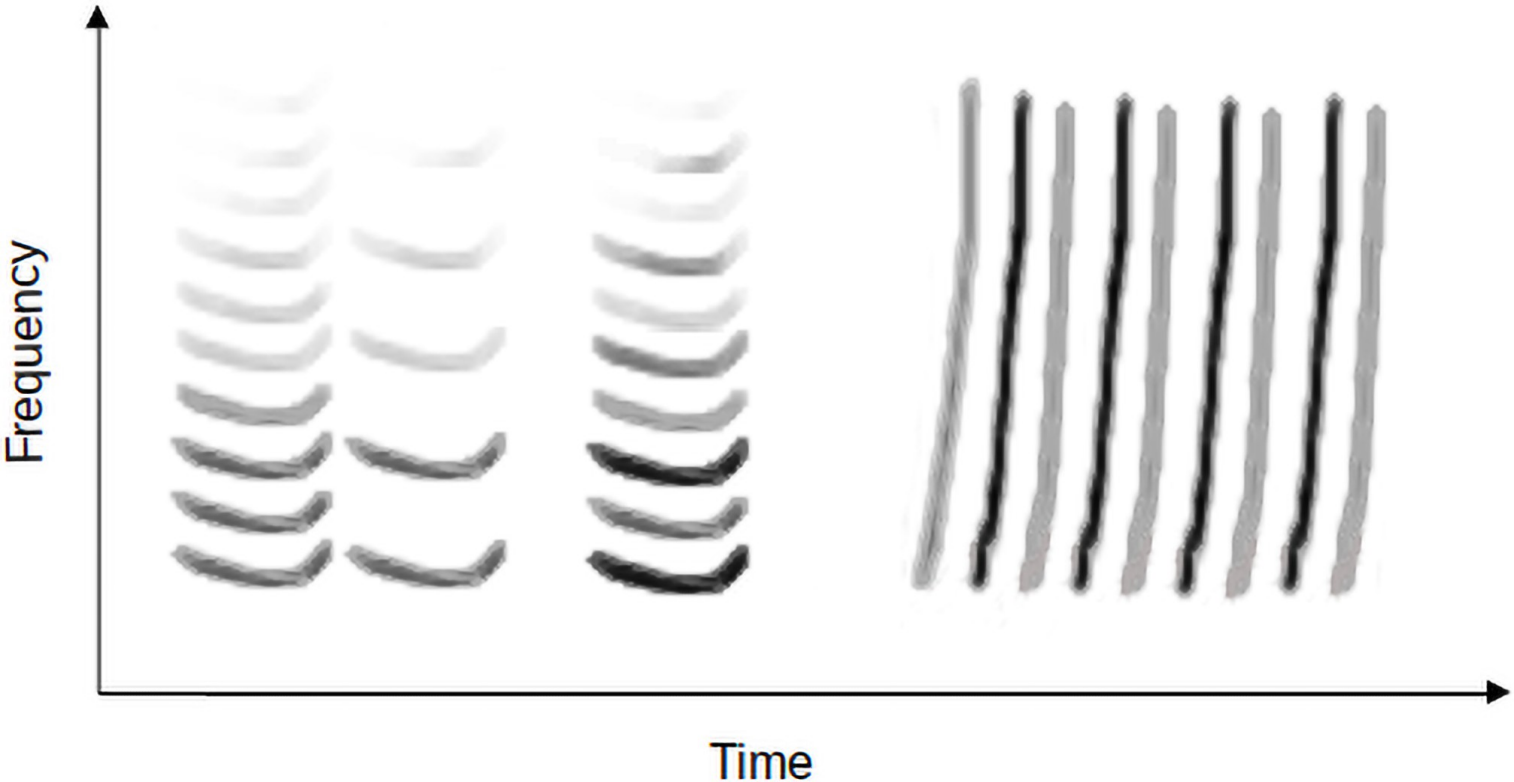
Acoustic reappearance diversity (in amplitude) also captures simple harmony and rhythm. A highly-simplified illustration of how *acoustic reappearance diversity* could be construed as a general enough construct to encapsulate aspects of both pitched and rhythmic musicality, despite the fact that it was not formulated using either. A pitched matched harmonic sound (**left**) with two different overlapping harmonic series (the higher frequency tone is bolded as it overlaps with the harmonics of the lower frequency tone one octave below it). A rhythmic pattern (**right**) with stresses, in bold, every other beat. This illustration is only a very simple demonstration of how our index could be expanded beyond the syllable or utterance level to incorporate higher system-level universals. In the example above, it is expanded to include reappearing diversity of *amplitude* across both frequency (left) and time (right). The reappearance diversity index could also conceivably be expanded to include much higher-order and complex attributes such as musical motif patterning or song repertoire typicality. It is important to reemphasize here, that despite its apparent redundancy with complexity, ARDI exceeds mere spectral shape enumeration (i.e. syllable count) by also requiring temporal or spectral patterning.

The six select structural acoustic features [34] that we found in primate call spectrograms, when explored together using PCA, helped us formulate a metric that emphasizes two contrasting forms of musical redundancy (see Fig 4: PC2). These forms, both spectral (e.g. transposition) and temporal (e.g. repetition), were combined as *reappearance* (Eq 1b). After multiplication by our *diversity* measure of syllable count, the resulting *reappearance diversity* [ARDI] index can serve as an estimate of the number of unique units that typically reappear within a call (Eq 2c). This formulation ensures that a call contains non-negligible amounts of both of these balancing and opposing musical forces. Although higher-level features [51, 52], such as those found in more rhythmic and tonal music, were ruled out by PCA—our *acoustic reappearance diversity* formulation could be reinterpreted to capture these anyway (see Fig 8). This definitional focus on the inter-unit relationships of spectral gestures in short duration acoustic displays allows for not only broad inclusion across taxa, but has uncovered many new primates with short, music-like calls including several species of monkey, lemur, and galago (Fig 10). Furthermore our definition is not delimited by non-acoustic universals such as mode of generation or context, and therefore better allows researchers to analyze them separately as evolutionary influences.

ARDI appears to capture song-like calls in primates more efficiently than traditional bird song measures such as raw syllable count (Fig 6, Table 3) but fails to associate highly with chorusing (Fig 7a). Rather than being a drawback of the index, however, lower ARDI scores for (inherently groupish) chorusing could instead be nevertheless interpreted as undermining any major group size selection effect for numerous performers, at least in non-human primates—as solitary species have higher than expected ARDI scores (Fig 8b). The fact that foraging calls scored reasonably high is not only supportive of AAH but is compatible with the view that more solitary primates could have been some of the first to evolve simple musical calls. Loud calls consisting of an accelerando temporal progression are thought to be an ancestral call morphotype for hominoids and perhaps even old world monkeys [4]. But our data hint at a possibly deeper origin via more subtly melodic locational calls, evidenced by the high ARDI scoring notes, twitters, chirps, and trills of galagos, tarsiers, titis, and gibbons—species representing disparate basal clades that all exhibit short transpositional progressions in their displays [29]. Perhaps the need for more efficient arboreal orientating between physically non-adjacent but neighborhood cohabiting primates, rather than the need for social display in larger group contexts, was a more likely instigator of ancient primate musicality.

We have presented the case for formulating ARDI—a continuous metric for musical behavior—to enable a gradualistic bridging of the evolutionary gap between human and animal musicality. Our simplified utterance-level index shows a strong association with the salient calls of small, family-sized groups [145]. But more sophisticated definitions of music—those more focused upon system-level features—may prove to correspond with more gregarious selective influences [146] such as collective action [31, 147] for more coordination during group hunting [148], or for maintaining contact during long-distance foraging or scavenging [44]. Even higher order structures (e.g. recursive and nested structures) could correlate with grammatical linguistic precursors [62, 149] or melodic intonations in modern languages. Accordingly, we surmise that human musicality likely evolved through a gradual accretion of features [47] derived from an existing substrate [64], where former adaptations are invariably re-purposed via different adaptive pressures into new functionality [47]. We concur with Darwin’s general observation, that, for humans, this existing substrate might have consisted of duet-like displays of our basal hominoid antecedents [5, 137]. And manifestations of modern primate musicality, however sparse, could have evolved through a gradual accretion of features [6, 42, 47] derived from transpositionally discerning calls [126] of more solitary and ancient prosimians and anthropoids.

## Supporting information

S1 Fig Avian example spectrograms used for feature scoring training. Tone, interval, rhythm (top row), repetition, transposition, and syllable count (bottom row) from low (left) to high (right) within each series. The syllable counts (bottom right) are: 1, 2, 3, 3, 3, 4 (approximately).
(TIF)

S2 Fig Boxplots of average pairwise differences between scorer combinations. First all possible 10 pairs of 5 scorers’ scores were subtracted from one another (**top**). Then the means of pairs were subtracted from means of the remainder triads to generate differences at these higher level aggregates (**bottom**). The contrast between top and bottom plot series demonstrates that while individual scores may not be extremely *individually* reliable, their *aggregated* (mean) values, between multiple scorers, are substantially less variable.
(TIF)

S1 File Reference list for spectrographic sources. This reference list is also mirrored at https://osf.io/bvsfz/.
(DOC)

S2 File Spectrogram scoring protocol. This protocol is also mirrored at http://doi.org/10.17504/protocols.io.bp5emq3e.
(DOC)

S3 File Code validating an approximation of the song complexity index. (DOC)

S1 Table (TIFF)
