# Supplementary figures and images for: On reappearance and complexity in musical calling
Source: PLoS One. 2021 Dec 17;16(12):e0218006. doi: 10.1371/journal.pone.0218006 (PMC8683036; doi:10.1371/journal.pone.0218006)

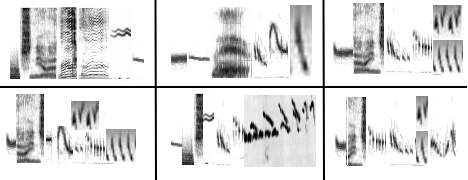

Supplement: S1 Fig — Tone, interval, rhythm (top row), repetition, transposition, and syllable count (bottom row) from low (left) to high (right) within each series. The syllable counts (bottom right) are: 1, 2, 3, 3, 3, 4 (approximately). (TIF) [file pone.0218006.s001.tif]

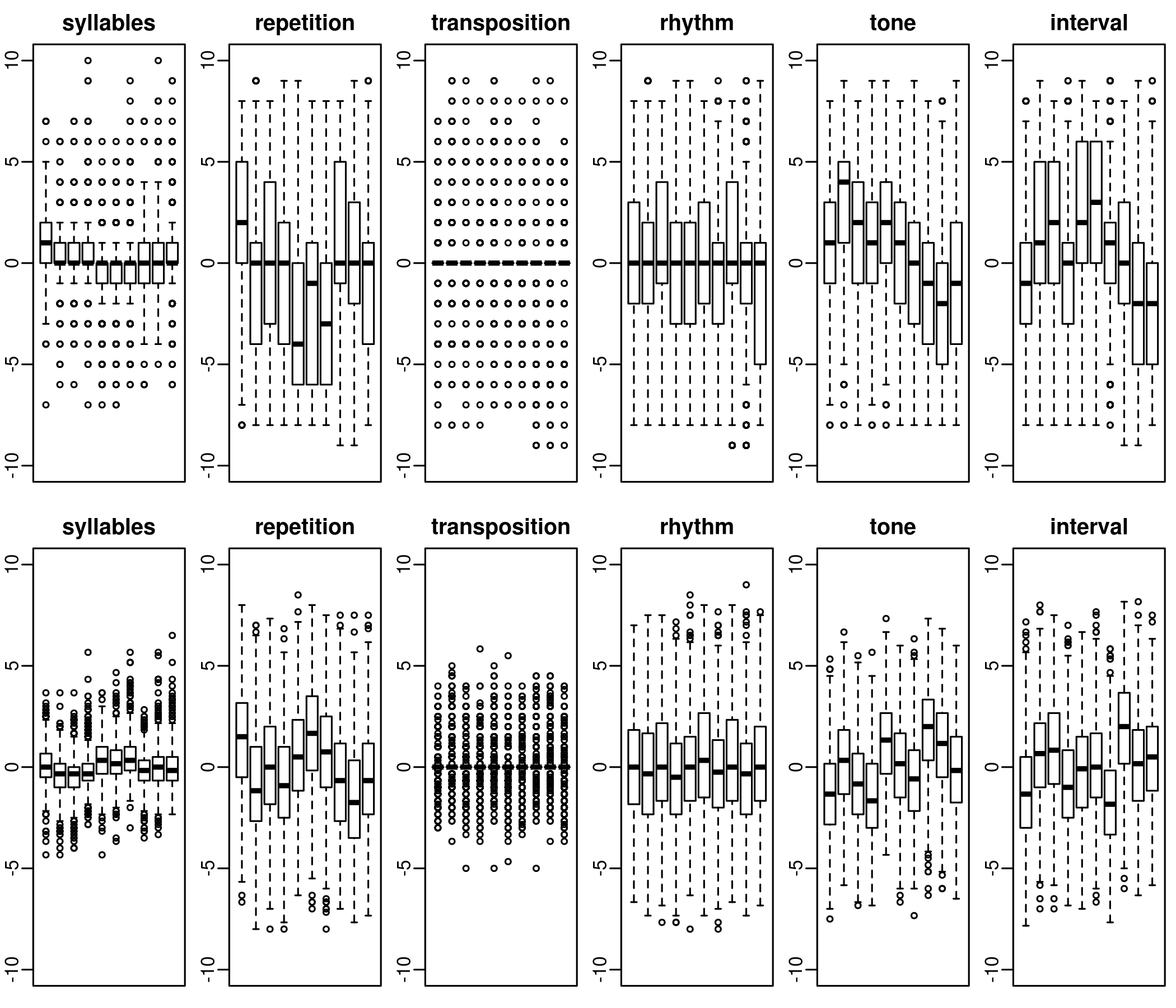

Supplement: S2 Fig — First all possible 10 pairs of 5 scorers’ scores were subtracted from one another (top). Then the means of pairs were subtracted from means of the remainder triads to generate differences at these higher level aggregates (bottom). The contrast between top and bottom plot series demonstrates that while individual scores may not be extremely individually reliable, their aggregated (mean) values, between multiple scorers, are substantially less variable. (TIF) [file pone.0218006.s002.tif]

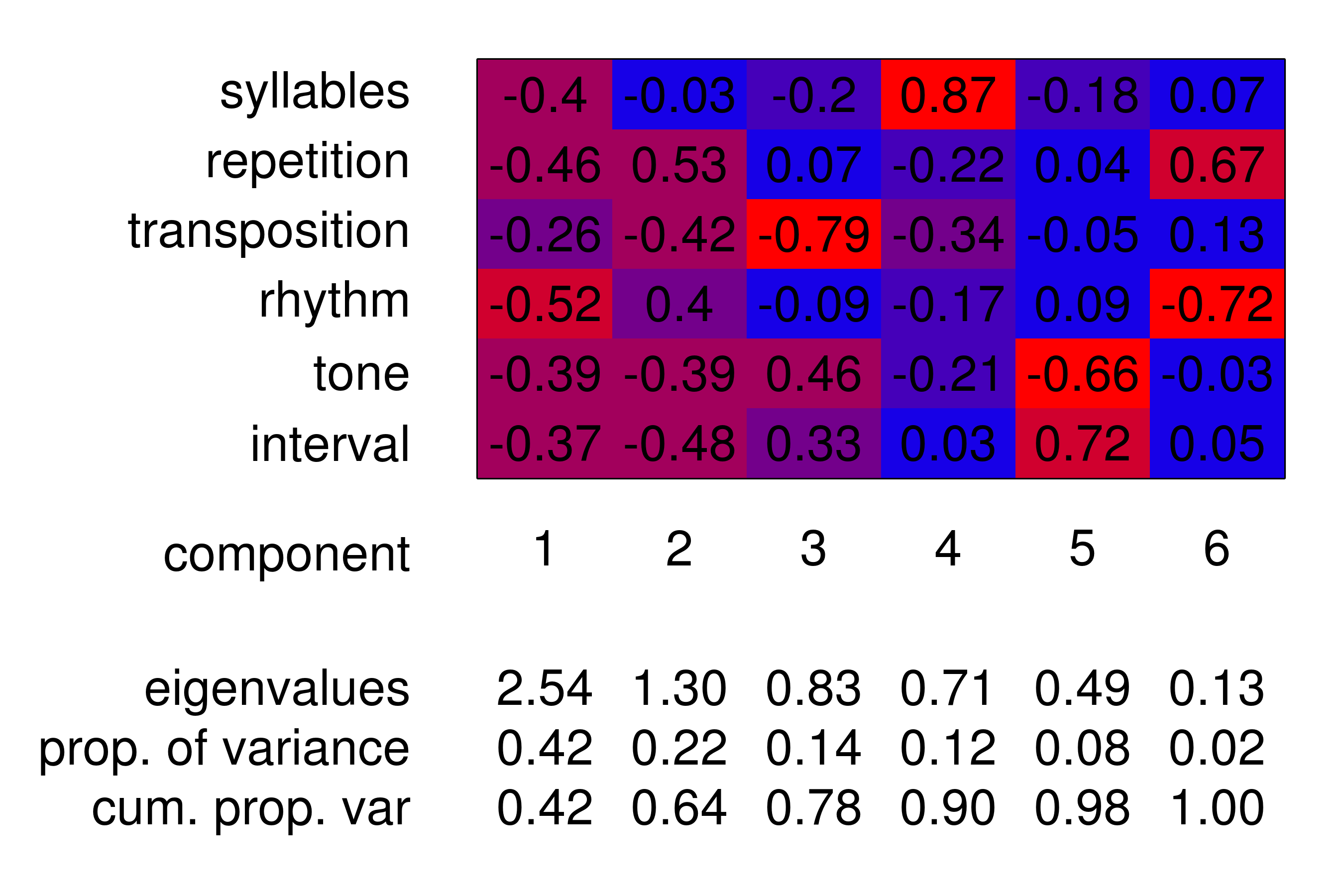

Supplement: S1 Table — (TIFF) [file pone.0218006.s006.tiff]
