# Supplementary material for: On reappearance and complexity in musical calling
Source: PLoS One. 2021 Dec 17;16(12):e0218006. doi: 10.1371/journal.pone.0218006 (PMC8683036; doi:10.1371/journal.pone.0218006)
Supplement: S1 File — This reference list is also mirrored at https://osf.io/bvsfz/. (DOC) [file pone.0218006.s003.doc]

**S1 File**

**Primate Vocalizations**

Řeháková-Petrů, M., Policht, R., and Peške, L. (**2012**). “Acoustic repertoire of the Philippine Tarsier (*Tarsius syrichta fraterculus*) and individual variation of long-distance calls,” Int. J. Zool. [DOI: 10.1155/2012/602401](https://doi.org/10.1155/2012/602401)

Aich, H., Moosheilen, R., and Zimmermann, E. (**1990**). “Vocalizations of adult Gelada Baboons (*Theropithecus-gelada*) - acoustic structure and behavioral context,” Folia Primatol. (Basel), **55**, 109–132. [DOI: 10.1159/000156508](https://doi.org/10.1159/000156508)

Baldwin, J. D., and Baldwin, J. I. (**1976**). “Vocalizations of Howler monkeys (Alouatta-palliata) in southwestern Panama,” Folia Primatol. (Basel), **26**, 81–108. [DOI: 10.1159/000155733](https://doi.org/10.1159/000155733)

Bermejo, M., and Omedes, A. (**1999**). “Preliminary vocal repertoire and vocal communication of wild bonobos (*Pan paniscus*) at Lilungu (Democratic Republic of Congo),” Folia Primatol. (Basel), **70**, 328–357. [DOI: 10.1159/000021717](https://doi.org/10.1159/000021717)

Bouchet, H., Blois-Heulin, C., and Lemasson, A. (**2012**). “Age- and sex-specific patterns of vocal behavior in De Brazza’s monkeys (*Cercopithecus neglectus*),” Am. J. Primatol., **74**, 12–28. [DOI: 10.1002/ajp.21002](https://doi.org/10.1002/ajp.21002)

Buchanan, D. (**1978**). *Communication and ecology of pithecine monkeys with special reference to Pithecia pithecia* Wayne State University, Detroit.

Casamitjana, J. (**2002**). “The vocal repertoire of the woolly monkey *Lagothrix lagothricha*,” Bioacoustics, **13**, 1–19. [DOI: 10.1080/09524622.2002.9753483](https://doi.org/10.1080/09524622.2002.9753483)

Charles-Dominique, P. (**1977**). *Ecology and behaviour of nocturnal primates: prosimians of equatorial West Africa*, Duckworth, London. [ISBN: 9780231043625](https://biblio.com/9780231043625)

Cherry, J. A., Izard, M. K., and Simons, E. L. (**1987**). “Description of ultrasonic Vocalizations of the Mouse Lemur (Microcebus-murinus) and the Fat-Tailed Dwarf Lemur (*Cheirogaleus-medius*),” Am. J. Primatol., **13**, 181–185. [DOI: 10.1002/ajp.1350130208](https://doi.org/10.1002/ajp.1350130208)

Clarke, E., Reichard, U. H., and Zuberbuhler, K. (**2006**). “The syntax and meaning of wild gibbon songs,” PLoS ONE, **1**, e73. [DOI: 10.1371/journal.pone.0000073](https://doi.org/10.1371/journal.pone.0000073)

Cleveland, J., and Snowdon, C. T. (**1982**). “The complex vocal repertoire of the adult Cotton-Top Tamarin (*Saguinus-oedipus-oedipus*),” Z. Tierpsychol.-J. Comp. Ethol., **58**, 231–270. [DOI: 10.1111/j.1439-0310.1982.tb00320.x](https://doi.org/10.1111/j.1439-0310.1982.tb00320.x)

Crockford, C., and Boesch, C. (**2005**). “Call combinations in wild chimpanzees,” Behaviour, **142**, 397–421. [DOI: 10.1163/1568539054012047](https://doi.org/10.1163/1568539054012047)

Ey, E. (**2008**). *Influences of ecological factors on vocal communication in olive baboons (Papio hamadryas anubis)* ([Ph.D. Thesis](https://www.uni-goettingen.de/en/dissertations/425425.html)), Georg-August University, Gottingen.

Fernandes, M. E. B. (**1991**). “Social communication of chiropotes (*Chiropotes satanas utahicki*, Cebidae, Primates) in captivity,” Primatol. No Bras., **3**, 296. [ISBN: 9788585401023](https://www.sbprimatologia.org.br/a-sbpr/publicacoes/a-primatologia-no-brasil/)

Fichtel, C. & Kappeler, P., M. (**2011**). Variation in the Meaning of Alarm Calls in Verreaux’s and Coquerel’s Sifakas (Propithecus verreauxi, P. coquereli). Int. J. Primatol. **32**, 346–361.

[DOI: 10.1007/s10764-010-9472-9](https://doi.org/10.1007/s10764-010-9472-9)

Fischer, J., Hammerschmidt, K. (**2002**). “An overview of the Barbary Macaque, Macaca sylvanus, Vocal Repertoire” Folia Primatol., **73**, 32-45. [DOI: 10.1159/000060417](https://doi.org/10.1159/000060417)

Fontaine, R. (**1981**). “The Uakaris, genus Cacajao,” In: Coimbra-Filho AF, Mittermeier RA, editors. Ecol. Behav. Neotropical Primates, Rio de Janeiro: Academia Brasileira de Ciencias.

Gamba, M., and Giacoma, C. (**2007**). “Quantitative acoustic analysis of the vocal repertoire of the crowned lemur,” Ethol. Ecol. Evol., 19:323-343. [DOI: 10.1080/08927014.2007.9522555](https://doi.org/10.1080/08927014.2007.9522555)

Geissmann, T., Nguyen, X. D., Lormee, N., and Momberg, F. (**2000**). *Vietnam primate conservation status review 2000, part 1: gibbons* Fauna & Flora International, Hanoi: Indochina Programme Office. <http://www.gibbons.de/main/books/2000GibbonStatusReview.pdf>

Geissmann, T. (**2000**). “Duet Songs of the Siamang, Hylobates Syndactylus: I. Structure Organization.” Primate Report, **56**, pp. 33–60. <http://www.gibbons.de/main/papers/pdf_files/2000duetstructure.pdf>

Gittins, S. P. (**1984**). “The vocal repertoire of the agile gibbon,” Lesser Apes Evol. Behav. Biol., Edinburgh University Press, Edinburgh. [ISBN: 9780852244487](https://biblio.com/9780852244487)

Gosset, D., Fornasieri, I., and Roeder, J. J. (**2000**). “Acoustic structure and contexts of emission of vocal signals by black lemurs,” Evol. Commun., **4,**  p. 225-251. [DOI: 10.1075/eoc.4.2.06gos](https://doi.org/10.1075/eoc.4.2.06gos)

Green, S. (**1975**). “Variation of vocal pattern with social situation in the Japanese monkey (*Macaca fuscata*): a field study,” Primate Behav., Academic Press, New York, Vol. 4, pp. 1–102.

Grimm, R. J. (**1967**). “Catalogue of Sounds of Pigtailed Macaque (Macaca Nemestrina).” *Journal*

*of Zoology* 152: 361. [DOI: 10.1111/j.1469-7998.1967.tb01650.x](https://doi.org/10.1111/j.1469-7998.1967.tb01650.x)

Gros-Louis, J. (**2002**). “Contexts and behavioral correlates of trill vocalizations in wild white-faced Capuchin monkeys (*Cebus capucinus*),” Am. J. Primatol., **57**, 189–202. [DOI: 10.1002/ajp.10042](https://doi.org/10.1002/ajp.10042)

Harcourt, A. H., Stewart, K. J., and Hauser, M. (**1993**). “Functions of wild gorilla close calls 1 repertoire, context, and interspecific comparison,” Behaviour, **124**, 89–122. [DOI: 10.1163/156853993X00524](https://doi.org/10.1163/156853993X00524)

Hohmann, G. (**1989**). “Vocal communication of wild Bonnet Macaques (*Macaca-radiata*),” Primates, **30**, 325–345. [DOI: 10.1007/BF02381258](https://doi.org/10.1007/BF02381258)

Hohmann, G. M., and Herzog, M. O. (**1985**). “Vocal communication in Lion-Tailed Macaques (*Macaca-silenus*),” Folia Primatol. (Basel), **45**, 148–178. [DOI: 10.1159/000156226](https://doi.org/10.1159/000156226)

Kudo, H. (**1987**). “The Study of Vocal Communication of Wild Mandrills in Cameroon in Relation to Their Social-Structure,” Primates, **28**, 289–308. [DOI: 10.1007/BF02381013](https://doi.org/10.1007/BF02381013)

Macedonia, J. M. (**1993**). “The vocal repertoire of the Ringtailed Lemur (*Lemur catta*),” Folia Primatol. (Basel), **61**, 186–217. [DOI: 10.1159/000156749](https://doi.org/10.1159/000156749)

Macedonia, J. M., and Stanger, K. F. (**1994**). “Phylogeny of the Lemuridae revisited - evidence from communication signals,” Folia Primatol. (Basel), **63**, 1–43. [DOI: 10.1159/000156787](https://doi.org/10.1159/000156787)

Mackinnon, J. (**1974**). “Behavior and Ecology of Wild Orangutans (*Pongo-Pygmaeus*),” Anim. Behav., **22**, 3–74. [DOI: 10.1016/S0003-3472(74)80054-0](https://doi.org/10.1016/S0003-3472(74)80054-0)

MacLanahan, E. B., and Green, K. M. (**1977**). “The vocal repertoire and an analysis of the contexts of vocalizations in *Leontopithecus rosalia*,” Biol. Conserv. Callitrichidae, Smithsonian Institute, Washington, DC, pp. 251–269. [ISBN: 9780874745863](https://biblio.com/9780874745863)

Maretti, G., Sorrentino, V., Finomana, A., Gamba, M., and Giacoma, C. (**2010**). “Not just a pretty song: an overview of the vocal repertoire of *Indri indri*,” [J. Anthropol. Sci.](https://www.jass-anthropology.com/contents/volume88-2010#h.p_1a9kYq1TtWzy), **88**, 151–165.

Masataka, N. (**1982**). “A field study on the vocalizations of Goeldi’s monkeys (*Callimico goeldii*),” Primates, **23**, 206–219. [DOI: 10.1007/BF02381161](https://doi.org/10.1007/BF02381161)

Mendez-Cardenas, M. G., and Zimmermann, E. (**2009**). “Duetting-a mechanism to strengthen pair bonds in a dispersed pair-living primate (*Lepilemur edwardsi*)?,” Am. J. Phys. Anthropol., **139**, 523–532. [DOI: 10.1002/ajpa.21017](https://doi.org/10.1002/ajpa.21017)

Moody, M. I., and Menzel, E. W. (**1976**). “Vocalizations and their behavioral contexts in tamarin *Saguinus-fuscicollis*,” Folia Primatol. (Basel), **25**, 73–94. [DOI: 10.1159/000155708](https://doi.org/10.1159/000155708)

Newman, J. D. (**1985**). “Squirrel monkey communication,” Handb. Squirrel Monkey Res., Plenum Press, New York, pp. 99–126. [ISBN: 9780306417542](https://biblio.com/9780306417542)

Nietsch, A. (**2003**). “Outline of the vocal behavior of *Tarsius spectrum*: call features, associated behaviors, and biological functions,” Tarsiers Past Present Future, 196-120, Rutgers University Press. [ISBN: 9780813532363](https://biblio.com/9780813532363)

Ouattara, K., Lemasson, A., and Zuberbühler, K. (**2009**). “Campbell’s Monkeys use affixation to alter call meaning,” (A. F. Y. Poon, Ed.) PLoS ONE, **4**, e7808. [DOI: 0.1371/journal.pone.0007808](https://doi.org/10.1371/journal.pone.0007808)

Palombit, R. A. (**1992**). “A preliminary study of vocal communication in wild Long-Tailed Macaques (*Macaca-fascicularis*) 1 vocal repertoire and call emission,” Int. J. Primatol., **13**, 143–182. [DOI: 10.1007/BF02547839](https://doi.org/10.1007/BF02547839)

Pereira, M. E., Seeligson, M. L., and Macedonia, J. M. (**1988**). “The behavioral repertoire of the Black-and-White Ruffed Lemur, *Varecia-variegata-variegata* (Primates, Lemuridae),” Folia Primatol. (Basel), **51**, 1–32. [DOI: 10.1159/000156353](https://doi.org/10.1159/000156353)

Pola, Y. V., and Snowdon, C. T. (**1975**). “Vocalizations of Pygmy Marmosets (*Cebuella-Pygmaea*),” Anim. Behav., **23**, 826–842. [DOI: 10.1016/0003-3472(75)90108-6](https://doi.org/10.1016/0003-3472(75)90108-6)

Raemaekers, J. J., Raemaekers, P. M., and Haimoff, E. H. (**1984**). “Loud Calls of the gibbon (*Hylobates-lar*) - repertoire, organization and context,” Behaviour, **91**, 146–189. [DOI: 10.1163/156853984X00263](https://doi.org/10.1163/156853984X00263)

Range, F., and Fischer, J. (**2004**). “Vocal repertoire of sooty mangabeys (*Cercocebus torquatus atys*) in the Tai National Park,” Ethology, **110**, 301–321. [DOI: 10.1111/j.1439-0310.2004.00973.x](https://doi.org/10.1111/j.1439-0310.2004.00973.x)

Robinson, J. G. (**1979**). “Analysis of the organization of vocal communication in the Titi Monkey *Callicebus-moloch*,” Z. Tierpsychol.-J. Comp. Ethol., **49**, 381–405. [DOI: 10.1111/j.1439-0310.1979.tb00300.x](https://doi.org/10.1111/j.1439-0310.1979.tb00300.x)

Robinson, J. G. (**1984**). “Syntactic structures in the vocalizations of Wedge-Capped Capuchin Monkeys, *Cebus-olivaceus*,” Behaviour, **90**, 46–79. [DOI: 10.1163/156853984X00551](https://doi.org/10.1163/156853984X00551)

Schel, A. M., and Zuberbühler, K. (**2012**). “Predator and non-predator long-distance calls in Guereza colobus monkeys,” Behav. Processes, **91**, 41–49. [DOI: 10.1016/j.beproc.2012.05.004](https://doi.org/10.1016/j.beproc.2012.05.004)

Schulze, H., and Meier, B. (**1995**). “Behavior of captive *Loris tardigradus nordicus*: A qualitative description, including some information about morphological bases of behavior,” Creat. Dark Noct. Prosimians, Plenum Press, New York. [ISBN: 9780306451836](https://biblio.com/9780306451836)

Stanger, K. F., and J. M. Macedonia (**1994**). “Vocalizations of Aye-Ayes (Daubentonia-

Madagascariensis) in Captivity.” *Folia Primatologica* **62**, 160–69. [DOI: 10.1159/000156773](https://doi.org/10.1159/000156773)

Struhsaker, T. T. (**1967**). “Auditory communication among vervet monkeys (*Cercopithecus aethiops*),” Soc. Commun. Primates, University of Chicago Press, Chicago, IL, pp. 281–324. [ISBN: 9780226016009](https://biblio.com/9780226016009)

Struhsaker, T. T. (**1975**). *The red colobus monkey*, University of Chicago Press, Chicago, IL. [ISBN: 9780226777696](https://biblio.com/9780226777696)

Tenaza, R. R., Fitch, H. M., and Lindburg, D. G. (**1988**). “Vocal behavior of captive Sichuan Golden Monkeys (*Rhinopithecus-r-roxellana*),” Am. J. Primatol., **14**, 1–9. [DOI: 10.1002/ajp.1350140102](https://doi.org/10.1002/ajp.1350140102)

Ybarra, M. A. S. (**1986**). “Loud calls of adult male Red Howling Monkeys (*Alouatta-seniculus*),” Folia Primatol. (Basel), **47**, 204–216. [DOI: 10.1159/000156278](https://doi.org/10.1159/000156278)

Zimmermann, E. (**1985**). “The vocal repertoire of the adult Senegal Bushbaby (*Galago-senegalensis-senegalensis*),” Behaviour, **94**, 212–233. [DOI: 10.1163/156853985X00190](https://doi.org/10.1163/156853985X00190)

Zimmermann, E. (**1985**). "Vocalizations and Associated Behaviors in Adult Slow Loris (Nycticebus,

Coucang)." Folia Primatologica 44(1): 52-64. [DOI: 10.1159/000156197](https://doi.org/10.1159/000156197)
