# Supplementary material for: On reappearance and complexity in musical calling
Source: PLoS One. 2021 Dec 17;16(12):e0218006. doi: 10.1371/journal.pone.0218006 (PMC8683036; doi:10.1371/journal.pone.0218006)
Supplement: S2 File — This protocol is also mirrored at http://doi.org/10.17504/protocols.io.bp5emq3e. (DOC) [file pone.0218006.s004.doc]

**S2 File**

**Spectrogram Scoring Protocol**

**For trainer**

**set-up**

1. sample spectrographic repertoires from the literature (as described in methods above)

2. screen capture or scan each figure and name it systematically

e.g. genus-species-voc#-name-author-year-figure.png

3. generate a list of vocalizations (in rows) from literature for recording info above (in columns)

4. give each vocalization a globally unique identifier (e.g. 3 digits for hundreds of calls)

5. create a scoring table for each of your scorers using the following:

a. use the list of vocalizations but add a uniquely randomized score id column

b. sort the table by this new, randomized score id column.

c. add feature columns across the top for structural acoustic features you would like to score

d. hide the columns relating to genus, species, name, author, & year

6. use these new scoring tables to generate a new directories for each vocalization (titled by score id)

7. copy the spectrographic figures associated with each of these vocalizations into each directory

**training:**

1. gather training materials from literature and reference texts; make these tangible via screenshots or

via print outs of definitions (S1 Table) and example spectrograms (see S1 Fig below)

2. post these on screen at a computer lab where training and scoring take place

3. pass out printed or digital copies of these for reference during the remainder of the scoring session

4. answer any clarifying questions before the session starts

**For scorer**

1.read the instructions for scoring (S1 Table) as well as printed or digital definitions

2. iterate through each vocalization (do each of the following for each vocalization in your spreadsheet)

a. rate, on a scale of 1-10, how tonal, intervalic, rhythmic, etc. each vocalization type is and enter it

b. count the number of different types of syllables or shapes and enter this in the syllables column

**S1 Fig. Avian example spectrograms used for feature scoring training.** Tone, interval, rhythm (top row), repetition, transposition, and syllable count (bottom row) from low (left) to high (right). The syllable counts (bottom right) are: 1, 2, 3, 3, 3, 4 (approximately)

**S1 Table. Vocalization spectrogram component definitions and scoring key.**

Listed here are our six structural acoustic features universal to human music, their definitions, and a spectrographically relevant interpretation for scoring purposes. The first five dimensions were scored on a scale of 1 (lowest) to 10 (highest), while syllable was scored as a count of different spectral shapes. This matrix, along with pictures of the original dictionary [88] and encyclopedia [87] definitions, and a small set of non-primate spectrographic examples (S1 Fig), constituted the entirety of materials provided to scorers during the hour-long computer lab training session. These features, which *themselves* proved difficult to impartially define, were verified using additional dictionaries [90] and encyclopedias [89]. While these feature scoring criterion err on the side of being too open-ended, we tried to give scorers as much free reign over the responses as possible so as to avoid our expectations from biasing these blind recruits. Furthermore, although a smaller scale (e.g. 1-5 rather than 1–10) could have been used, we desired high rather than low resolution raw data where subsequent binning into fewer categories (e.g. binary) would still be possible anyway.

**Simulation code for comparing call versus unit levels of repetition for calculations of SCI**

This simulation suggests that there is only a somewhat negligible difference (~2%) between the repetition percentage based approximation for SCI versus the repeats summation version (S3 Fig).

# Below are two versions for calculating the song complexity index [SCI]:

sci_f <- function(n,l,reps){n * sqrt(l^2 - sum(sapply(reps,function(m)( m - 1)^2)))}

# above: original SCI; below: approximate SCI using call-level repetition instead

m_avg <- function(n,l,r){(l*r)/(n*r)} #ratio of repeated units to repeated (unique) syllables

sci_r <- function(n,l,r) {n * sqrt(l^2 - n*( m_avg(n,l,r) - 1)^2) }

### simulation to prove n*(m_avg(l,n,r) - 1)^2 ~= sum(sapply(reps,function(m)( m - 1)^2)

units_max <- 50/2

syllables_max <- 26/5

call_count <- 10000

unit_median <- 3

sim_n <- 5000

sci_fs <- rep(0,sim_n)

sci_rs <- rep(0,sim_n)

rep_facts <- rep(0,sim_n)

for(i in 1:sim_n){

# create new sequence of numbers and convert them to letters (both from uniform)

len=runif(n=1,min=1,max=units_max) # units in this

units <- round(runif(n=len,min=1,max=syllables_max))

seq <- sapply(units,FUN=function(x) letters[x])

f<-table(seq) # frequency table (counts per letter)

l <- length(seq) # total letters

n <- length(f) # number of unique letters

r <- sum(f>1)/n # repetition percentage = number of repeated letters / all letters

rep_facts[i] <- r

#calculate CSI with

sci_fs[i] <- sci_f(n,l,f) # n*(E(m)-1)

sci_rs[i] <- sci_r(n,l,r) # sum(mi-1)

}

fit <- lm(sci_rs~sci_fs) # slope = .975

plot(sci_fs,sci_rs, col=rainbow(10)[rep_facts*10])

abline(fit, col='green', lwd=2)

abline(0,1, col='red', lty=3)
