# Supplementary material for: On reappearance and complexity in musical calling
Source: PLoS One. 2021 Dec 17;16(12):e0218006. doi: 10.1371/journal.pone.0218006 (PMC8683036; doi:10.1371/journal.pone.0218006)
Supplement: S3 File — (DOC) [file pone.0218006.s005.doc]

**Simulation code for comparing call versus unit levels of repetition for calculations of SCI**

This simulation suggests that there is only a somewhat negligible difference (~2%) between our repetition percentage (length-) based approximation for SCI versus the original, repeats summation version.

# Below are two versions for calculating the song complexity index [SCI]:

sci_f <- function(n,l,reps){n * sqrt(l^2 - sum(sapply(reps,function(m)( m - 1)^2)))}

# above: original SCI; below: approximate SCI using call-level repetition instead

m_avg <- function(n,l,r){(l*r)/(n*r)} #ratio of repeated units to repeated (unique) syllables

sci_r <- function(n,l,r) {n * sqrt(l^2 - n*( m_avg(n,l,r) - 1)^2) }

### simulation to prove n*(m_avg(l,n,r) - 1)^2 ~= sum(sapply(reps,function(m)( m - 1)^2)

units_max <- 50/2

syllables_max <- 26/5

call_count <- 10000

unit_median <- 3

sim_n <- 5000

sci_fs <- rep(0,sim_n)

sci_rs <- rep(0,sim_n)

rep_facts <- rep(0,sim_n)

for(i in 1:sim_n){

# create new sequence of numbers and convert them to letters (both from uniform)

len=runif(n=1,min=1,max=units_max) # units in this

units <- round(runif(n=len,min=1,max=syllables_max))

seq <- sapply(units,FUN=function(x) letters[x])

f<-table(seq) # frequency table (counts per letter)

l <- length(seq) # total letters

n <- length(f) # number of unique letters

r <- sum(f>1)/n # repetition percentage = number of repeated letters / all letters

rep_facts[i] <- r

#calculate CSI with

sci_fs[i] <- sci_f(n,l,f) # n*(E(m)-1)

sci_rs[i] <- sci_r(n,l,r) # sum(mi-1)

}

fit <- lm(sci_rs~sci_fs) # slope = .975

plot(x=sci_fs,y=sci_rs, col=rainbow(10)[rep_facts*10],

xlab='SCI (original)', ylab='SCI (approximation)')

abline(fit, col='green', lwd=2)

abline(0,1, col='red', lty=3)
